# Cannabis against chronic musculoskeletal pain: a scoping review on users and their perceptions

**DOI:** 10.1186/s42238-021-00096-8

**Published:** 2021-09-04

**Authors:** Daniela Furrer, Edeltraut Kröger, Martine Marcotte, Nathalie Jauvin, Richard Bélanger, Mark Ware, Guillaume Foldes-Busque, Michèle Aubin, Pierre Pluye, Clermont E. Dionne

**Affiliations:** 1 Centre d’excellence sur le vieillissement de Québec (CEVQ), Centre Intégré Universitaire de Santé et de Services Sociaux de la Capitale-Nationale (CIUSSSCN), Québec, QC Canada; 2 Centre de Recherche du CHU de Québec-Université Laval, Québec, QC Canada; 3 Faculty of Pharmacy, Université Laval, Québec, QC Canada; 4 Hôpital du Saint-Sacrement, 1050 Chemin Ste-Foy, room L2-30, Québec, QC G1S 4L8 Canada; 5 Faculty of Medicine, Université Laval, Québec, QC Canada; 6 Faculty of Medicine, McGill University, Montréal, QC Canada; 7 School of Psychology, Université Laval, Québec, QC Canada; 8 Research Centre of the Centre Intégré de Santé et de Services Sociaux (CISSS) de Chaudière-Appalaches, Lévis, QC Canada

**Keywords:** Medical cannabis, Musculoskeletal pain, Chronic pain, Non-cancer chronic pain, Perceived effects, Adverse effect

## Abstract

**Background:**

Chronic musculoskeletal pain (CMP) may lead to reduced physical function and is the most common cause of chronic non-cancer pain. Currently, the pharmacotherapeutic options against CMP are limited and frequently consist of pain management with non-steroidal anti-inflammatories, gabapentinoids, or opioids, which carry major adverse effects. Although the effectiveness of medical cannabis (MC) for CMP still lacks solid evidence, several patients suffering from it are exploring this therapeutic option with their physicians.

**Objectives:**

Little is known about patients’ perceptions of their MC treatment for CMP. We aimed to increase this knowledge, useful for healthcare professionals and patients considering this treatment, by conducting a scoping literature review, following guidance by Arksey and O’Malley, to describe the views and perceptions of adult patients who had consumed MC to relieve chronic CMP.

**Methods:**

Databases (PUBMED, EMBASE, Web of Science) and websites were searched using combinations of controlled and free vocabulary. All studies and study designs reporting on patients’ perceptions regarding MC against CMP were considered. Studies had to include adult patients reporting qualitatively or quantitatively, i.e., through questionnaires, on MC use to treat CMP or other non-cancer pain, since studies reporting exclusively on perceptions regarding CMP were very rare. Study characteristics were extracted and limitations of the study quality were assessed. The review includes patients’ demographic characteristics, patterns of MC use, perceived positive and negative effects, use of alcohol or other drugs, reported barriers to CM use, and funding sources of the studies.

**Results:**

Participants of the 49 included studies reported that MC use helped them to reduce CMP and other chronic non-cancer pain, with only minor adverse effects, and some reported improved psychological well-being.

In the included studies, men represent between 18 and 88% of the subjects. The mean age of participants in these studies (42/49) varied between 28.4 and 62.8 years old. The most common route of administration is inhalation.

**Conclusion:**

MC users suffering from CMP or other chronic non-cancer pain perceived more benefits than harms. However, the information from these studies has several methodological limitations and results are exploratory. These user-reported experiences must thus be examined by well-designed and methodologically sound clinical or observational studies, particularly regarding CMP, where reports are very scarce.

**Supplementary Information:**

The online version contains supplementary material available at 10.1186/s42238-021-00096-8.

## Background

### Musculoskeletal pain

Musculoskeletal pain is a condition affecting bones, muscles, ligaments, and joints, resulting from underlying diseases or health problems such as osteoarthritis, inflammatory rheumatic diseases, and fibromyalgia, although in many cases the exact cause cannot be identified (Arthritis Society, 2015a). Musculoskeletal pain is the most common type of severe long-term pain and it impacts on all aspects of life by typically affecting dexterity and mobility, and by limiting work and activities of daily living (Woolf et al., 2012). It has been recently reported that one in two American adults lives with a musculoskeletal disease (Yelin et al., 2016), and in Canada, approximately 17% of the adult population are affected, nearly half of whom (44%) are aged 65 years or older (Arthritis Society, 2015a). Some cases of musculoskeletal pain are of short duration and have no long-term consequences. Chronic musculoskeletal pain (CMP), which persists for more than 3 months (Task Force on Taxonomy of the International Association for the Study of Pain, 1994), however, is associated with a range of problems such as sleep disorders, depression, anxiety, fatigue, reduced quality of life, and inability to work or socialize (Moore et al., 2014). In the USA, the impact of CMP on the economy in terms of healthcare costs and lost productivity is estimated at US $304 billion for the year 2013 (Yelin et al., 2016).

Effective pharmacological therapeutic options for the relief of CMP are limited and the treatment remains suboptimal for many patients (Fitzcharles et al., 2016). Examples for this are the use of non-steroidal anti-inflammatories, gabapentinoids (e.g., pregabalin and gabapentin), or the antidepressants duloxetine and milnacipran, which have shown clinical efficacy in the treatment of fibromyalgia and may have benefit in osteoarthritis and low back pain. However, it is estimated that only about one-third of patients will have at least 50% pain relief with one of these agents used as monotherapy; due to significant adverse effects, patients often fail to achieve recommended doses, further diminishing the medications’ effectiveness (Goldenberg et al., 2011). Opioids are also used to manage CMP, although the effectiveness of this approach remains uncertain (Petzke & Enax-Krumova, 2016; Schaefert et al., 2015) and the clinical management of CMP with opioids is challenging due to adverse effects such as dependence and/or addiction leading to possible overdose and death (Atluri et al., 2014; Ballantyne, 2015; Hauser et al., 2016; Tobin et al., 2016). It is therefore urgent to explore new treatment options to relieve pain in persons affected by CMP and thus improve their quality of life and social participation (Rowe & Caprio, 2013; Gereau et al., 2014; Lynch & Ware, 2015). Many persons for whom CMP is not satisfactorily relieved are turning to alternative therapies. Among these, the products derived from cannabis are perceived as an interesting analgesic option, both by some physicians and some patients (Elikottil et al., 2009; Boehnke et al., 2016), although its use remains controversial (Hosking & Zajicek, 2008; D'Souza & Ranganathan, 2015).

### Cannabis and cannabinoids

The *Cannabis sativa* plant contains over 100 cannabinoids (ElSohly & Gul, 2014). The most abundant cannabinoid, delta-9-tetrahydrocannabinol (THC), is responsible for the main psychoactive effect of cannabis, but preclinical studies suggest that THC also has some analgesic and anti-inflammatory effects (Ashton, 2007). The second most abundant cannabinoid, cannabidiol (CBD), has antipsychotic effects and is not intoxicating (Niesink & van Laar, 2013; Zhu et al., 2006). Preclinical studies also support anti-inflammatory and analgesic effects of this compound (Burstein, 2015; Costa et al., 2007; Maione et al., 2011). The quantities and proportions of the different cannabinoids vary between different sources and preparations of cannabis (Ashton, 2001; de Meijer, 2014). Furthermore, there are differences between herbal preparations and consumption methods of cannabis regarding levels of individual cannabinoids, and between patients regarding the pharmacokinetics of these molecules (MacCallum & Russo, 2018). These differences affect treatment experiences (i.e., anxiety compared to relaxation), making it hard to come up with evidence-based information to guide physicians and patients on the most appropriate prescribing and dosing of cannabis for a given case (Beaulieu et al., 2016; Ko et al., 2016). Worldwide, several cannabinoid-based medicines are available in several countries. The first product, nabiximols (tradename Sativex®), contains the cannabinoids THC and CBD. The most common indication for its use is spasticity associated with multiple sclerosis. The second product, nabilone (tradename Cesamet®) contains a synthetic cannabinoid similar to THC and is used to alleviate nausea and vomiting associated with chemotherapy treatments. The third product, dronabinol (tradename Marinol®), is a synthetic cannabinoid chemically identical to THC and its main indications are anorexia associated with weight loss in patients with AIDS, as well as severe nausea and vomiting caused by cancer chemotherapy (Abuhasira et al., 2018). Quite recently, a product containing cannabidiol, Epidiolex®, has been approved by the US Food and Drug Administration for the treatment of Dravet syndrome and Lennox-Gastaut syndrome, which are severe epileptic encephalopathies.

### Medical cannabis and musculoskeletal pain: gaps in knowledge

Given the confusion between the terms cannabis, cannabinoids, and cannabis for medical purposes, we will refer to the term “medical cannabis” (MC) in this review, in order to describe cannabis products (plant-based products or pharmaceutical products) used for CMP or other non-cancer chronic pain. Chronic pain in general, including CMP, is the most common reason given for the therapeutic use of MC among adults (Fitzcharles et al., 2016; Swift et al., 2005; Ware et al., 2005; Aggarwal et al., 2009; Arthritis Society, 2015b). The effectiveness of MC in the management of such pain, however, remains controversial. In a systematic review and meta-analysis on cannabinoids for medical use by Whiting et al., only 4 of the 79 trials included were judged at low risk of bias (Whiting et al., 2015). Individual studies suggested improvement in pain intensity, but most of the differences did not reach clinical significance and there was no clear evidence for an effect of the type of cannabinoid or the mode of administration. It is also important to note that different products were used in the individual studies, plant based or pharmaceutical, making comparisons between the studies even more difficult. Moreover, none of the studies assessed the long-term effects of cannabinoids.

In 2015, Lynch et al. published a systematic review of randomized controlled trials published since 2010 and examining cannabinoids for the treatment of chronic non-cancer pain, including CMP. They reported that seven out of the 11 included studies demonstrated a significant analgesic effect. Several trials also demonstrated improvement in secondary outcomes (e.g., sleep, muscle stiffness, and spasticity) (Lynch & Ware, 2015). Adverse effects most frequently reported, such as fatigue and dizziness, were mild to moderate in severity and generally well tolerated.

In 2017, the National Academies for Science, Engineering, and Medicine of the USA published an exhaustive review on the health effects of cannabis and cannabinoids and concluded that “there is conclusive or substantial evidence that cannabis or cannabinoids are effective for the treatment of chronic pain in adults”, based on a review of reviews, following the conclusions of Whiting et al. (Whiting et al., 2015), as well as two primary studies (National Academies of Sciences E, and Medicine, 2017). It should be pointed out, however, that the conclusions reported in the paper of Whiting et al. should be regarded with caution, as most of the studies assessed in this systematic review showed a high risk of bias.

In 2018, Stockings et al. performed another systematic review and meta-analysis of 47 randomized controlled studies and 57 observational studies on cannabinoids for the treatment of chronic non-cancer pain and concluded that the evidence for the effectiveness of MC on chronic non-cancer pain is limited [pooled events rates for 50% reduction in pain were not significant: 18.2% (cannabinoids) vs 14.4% (placebo); moreover, the number needed to treat was high (NNT = 24; 95% CI: 15–61) and the number needed to harm was low (NNH = 6; 95% CI: 5–8)]. From the results of the reviewed studies, the authors considered it unlikely that cannabinoids would become an important treatment option in chronic non-cancer pain (Stockings et al., 2018). Similarly, Nugent et al. reported in their 2017 review that the utilization of MC to alleviate chronic pain might be associated with several harms, including increased risk for motor vehicle accidents, psychotic symptoms, and short-term cognitive impairment, in addition to negative impacts on the respiratory tract (Nugent et al., 2017).

Thus, available evidence on the effectiveness of MC against CMP and other chronic non-cancer pain remains limited and the results of systematic reviews are somewhat inconclusive. It is even more difficult to conclude about the use of cannabis specifically in the management of CMP because, according to three systematic reviews of clinical trials on cannabis (Fitzcharles et al., 2016; Stockings et al., 2018), only two clinical trials have focused exclusively on musculoskeletal conditions. The authors of these clinical trials reported that cannabinoids (nabilone or Sativex®) led to a significant decrease in some aspects of pain in patients with fibromyalgia (Skrabek et al., 2008) or rheumatoid arthritis (Blake et al., 2006). However, only a small number of patients were studied for a short period of time in these trials and further methodological limitations may have affected their quality (Aviram & Samuelly-Leichtag, 2017) (Fitzcharles et al., 2016; Stockings et al., 2018). In conclusion, more high-quality randomized controlled trials comparing herbal cannabis or pharmaceutical cannabinoids with established therapies or placebo are necessary to define their role in the management of CMP or other chronic pain (Fitzcharles et al., 2016).

Although the use of MC remains controversial, it is gaining popularity and legal frameworks for its use are increasingly seen under certain conditions in a growing number of countries, i.e., Australia, France, Israel, the Netherlands, the UK, New Zealand, Spain, Germany, 29 US states, and since 1999 in Canada (Aguilar et al., 2018), where “serious arthritis” was mentioned as one of the main diagnoses justifying a license to obtain cannabis for medical use in 2013 (Arthritis Society, 2015b). Several countries are therefore already confronted with increasing use of MC against CMP, including self-medication, even though its efficacy and safety are still unknown.

Two recent reviews reported on MC use in patients suffering from different diseases, including anxiety, depression, HIV/AIDS, pain, and multiple sclerosis, highlighting that pain is the most frequent reason for MC use and its increasing frequency in general and cannabis self-medication in particular (Kosiba et al., 2019; Park & Wu, 2017). However, we did not identify major reviews on the characteristics, motivations, perceptions, and expectations of patients with regard to the use of medical cannabis against musculoskeletal or other chronic non-cancer pain. Thus, a knowledge gap exists in our understanding of patients’ characteristics and perceptions with regard to this use. Therefore, we conducted a scoping review to explore and describe these characteristics and perceptions of persons using MC against chronic non-cancer pain, including CMP. This review represents a first step towards a larger research program on this topic.

## Methods

### Eligibility criteria and selection of articles

The study protocol was submitted to the funding organizations and can be accessed through the corresponding author. Included studies had to comprise adults having used cannabis or cannabinoids for therapeutic purposes, including CMP or other chronic pain. Moreover, study samples had to have included at least several participants with chronic musculoskeletal or non-cancer pain. Qualitative, quantitative, and mixed methods studies were considered.

Studies that were specific to only one disease, other than musculoskeletal conditions or chronic non-cancer pain, such as HIV/AIDS, cancer, multiple sclerosis, epilepsy, inflammatory bowel disease, glaucoma, Tourette’s syndrome, neuropathic pain, spinal cord injury, migraine, post-traumatic stress disorder, dementia, or mental illness, as well as palliative care, were excluded. Furthermore, all studies that did not report any patient perceptions or results—including clinical trials on the therapeutic or adverse effects of cannabis—were excluded. Books, meeting abstracts, editorials, letters, policy evaluations, or newspaper articles were also excluded. Initial eligibility was assessed by screening the titles and abstracts of retrieved references by three persons Daniela Furrer, Martine Marcotte, and Norma Perez. Then, full texts of eligible references were reviewed by three persons (Daniela Furrer, Martine Marcotte, and Rosa Martins). Included publications that reported about one study in two or more articles were combined into a single study, with one exception (see below). Thereafter, reference lists of relevant reviews and of included studies were hand searched for additional references following the same procedure.

### Information sources

Three large databases (MEDLINE, EMBASE, and Web of Science) were searched using keywords from the controlled vocabulary and free text, and combined to identify publications on users of cannabis for therapeutic purposes (see search strategies in Appendix 1). The searches were conducted during the second half of 2016, updated in June 2019, and were restricted to publications in English, French, or German with no other time limit.

### Search strategy

This scoping review followed guidance by Arksey and O’Malley, Levac et al., and Colquhoun et al. (Arksey & O'Malley, 2005; Levac et al., 2010; Colquhoun et al., 2014) and examined the published knowledge regarding perceptions and experiences of MC users suffering from CMP or chronic non-cancer pain. Early search results revealed the scarcity of publications studying MC users for CMP specifically, and since CMP represents the most common etiology for chronic non-cancer pain, we expanded our search to all studies including patients using MC for chronic non-cancer pain (Podichetty et al., 2003). Moreover, given the scarcity of studies on the perceptions of users of MC, we decided to include both plant-based products and pharmaceutical products such as nabilone or nabiximols in the present review, similarly to some of the included studies (Hazekamp et al., 2013). As such, in the remainder of the manuscript, the abbreviation MC refers to both plant-based products and cannabis-derived medicine.

### Data collection and quality appraisal

For this narrative synthesis, the following data were extracted by three persons into pre-determined Word files (Daniela Furrer, Martine Marcotte, and Rosa Martins) from the included studies: study design and setting, period of data collection, sample size, participants’ age and sex, indications for MC consumption, patterns of MC use, perceived benefits and adverse effects of use, and financial support for the study. When available, MC consumption as a substitute for other drugs, as well as barriers to MC use, were also documented. No individual quality appraisal was performed, according to the guidance used (Arksey & O'Malley, 2005; Levac et al., 2010; Colquhoun et al., 2014), but multiple limitations of the included study designs are outlined in the discussion.

## Results

**Fig. 1:**
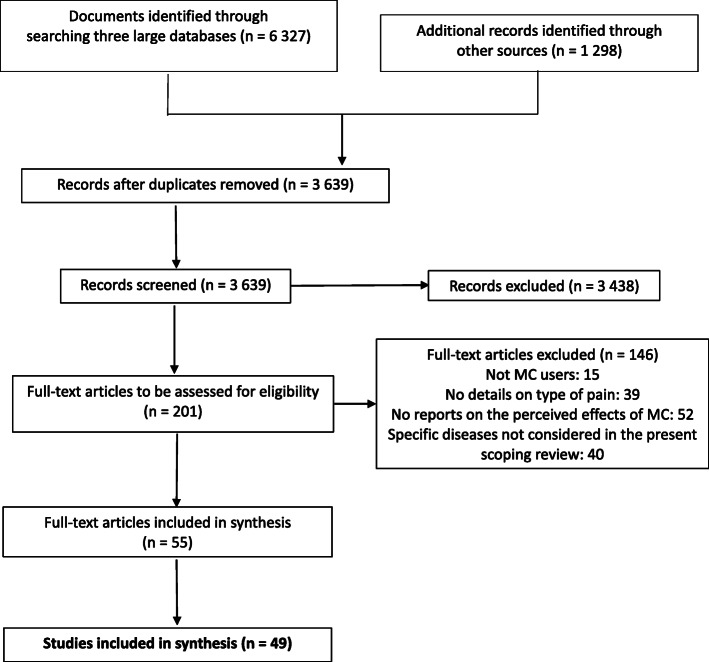
Flowchart of the scoping review

A total of 3639 references were first identified, and the full-text was screened for 201 articles, of which 52 publications reporting on 49 studies met the inclusion criteria (Fig. 1). In one publication (Perron et al., 2015), a sub-sample from a previous study (Ilgen et al., 2013) was used but, since study objectives and measures were different, they were treated as two different studies.

### Characteristics of the included studies

**Fig. 2:**
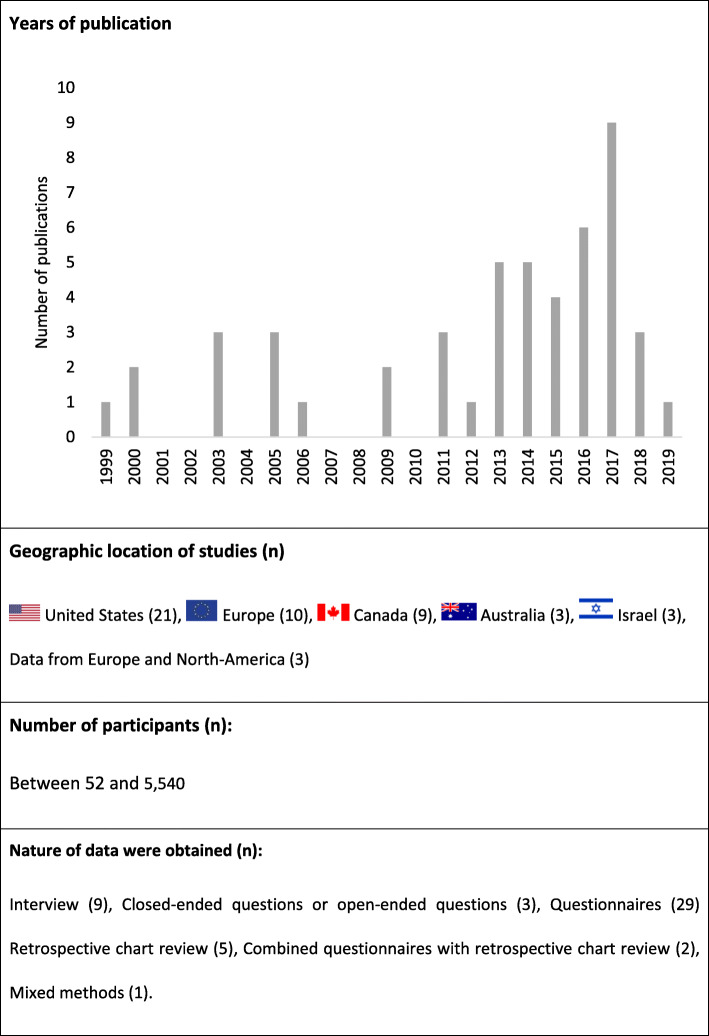
Medical cannabis and musculoskeletal pain: scoping review key data

The main characteristics of all included studies are summarized in Fig. 2.

Among all included studies, only two examined the prevalence of cannabis use exclusively among patients suffering from CMP (Ste-Marie et al., 2016). Most of the studies focused on mixed samples that included patients with CMP (between 2 and 91% of participants) (31 studies) (Swift et al., 2005; Aggarwal et al., 2009; Hazekamp et al., 2013; Ilgen et al., 2013; Aggarwal et al., 2013a; Aggarwal et al., 2013b; Belle-Isle et al., 2014; Bottorff et al., 2011; Bruce et al., 2018; Coomber et al., 2003; Degenhardt et al., 2015; Erkens et al., 2005; Gorter et al., 2005; Haroutounian et al., 2016; Harris et al., 2000; Hoffman et al., 2017; Kilcher et al., 2017; Lucas & Walsh, 2017; Lynch et al., 2006; Nunberg et al., 2011; Ogborne et al., 2000; Pedersen & Sandberg, 2013; Piper et al., 2017; Reinarman et al., 2011; Schnelle et al., 1999; Sexton et al., 2016; Shiplo et al., 2016; Ste-Marie et al., 2012; Troutt & DiDonato, 2015; Walsh et al., 2013; Ware et al., 2003) or experiencing unspecified chronic non-cancer pain (between 24 and 97% of participants) (17 studies) (Boehnke et al., 2016; Perron et al., 2015; Alexandre, 2011; Bonn-Miller et al., 2014; Brunt et al., 2014; Corroon Jr. et al., 2017; Cranford et al., 2016; Crowell, 2017; Fanelli et al., 2017; Grella et al., 2014; Grotenhermen & Schnelle, 2003; Hazekamp & Heerdink, 2013; Reiman, 2009; Reiman et al., 2017; Shah et al., 2017; Webb & Webb, 2014; Zaller et al., 2015).

### Funding

Funding information was reported in 28 of the 49 (57%) studies (Table 1); 23 studies were funded by research grants or governmental scholarships (Aggarwal et al., 2009; Perron et al., 2015; Ste-Marie et al., 2016; Aggarwal et al., 2013a; Aggarwal et al., 2013b; Belle-Isle et al., 2014; Bruce et al., 2018; Degenhardt et al., 2015; Erkens et al., 2005; Haroutounian et al., 2016; Harris et al., 2000; Hoffman et al., 2017; Lucas & Walsh, 2017; Pedersen & Sandberg, 2013; Sexton et al., 2016; Shiplo et al., 2016; Ste-Marie et al., 2012; Walsh et al., 2013; Brunt et al., 2014; Corroon Jr. et al., 2017; Cranford et al., 2016; Grella et al., 2014; Lavie-Ajayi & Shvartzman, 2018). Two studies were supported by non-governmental organizations (Hazekamp et al., 2013; Gorter et al., 2005). Five studies received mixed funding from research grants, non-governmental organizations, dispensaries or private foundations (Nunberg et al., 2011; Piper et al., 2017; Reinarman et al., 2011; Ware et al., 2003; Bonn-Miller et al., 2014; Lintzeris et al., 2018). Those five studies also had received funding from commercial cannabis interest or cannabis patient groups (Hazekamp et al., 2013; Gorter et al., 2005; Nunberg et al., 2011; Reinarman et al., 2011; Bonn-Miller et al., 2014; Lintzeris et al., 2018).

**Table 1:** Brief summary of included studies

Article	Study		Participants	Reasons for using cannabis medically	Reported effects and perceptions of medical cannabis	Funding
	Objectives/design: data source; recruitment	Location /period, legality^**1**^	Number/age/sex			
Aggarwal et al. 2009	To characterize chronic pain patients seeking medical cannabis treatment. Quantitative: Retrospective chart review; recruitment via a regional pain clinic.	Washington State, USA. 2007–2008, study, access points for medical cannabis dispensing in urban centers were informally tolerated.	139 patients seeking treatment with medical cannabis. Median 47 (18–84) years. 63% men.	Chronic pain: 82% myofascial pain syndrome 64% neuropathic pain 27% osteoarthritis.	The majority of patient records documented significant symptom alleviation.	Scholarship funding *National Institute of General Medical Sciences of the NIH *National Science Foundation
Aggarwal et al. (2013a & 2013b)	To present data from a dispensary-based survey of medical cannabis users. Quantitative: Dispensary-based survey; recruitment through an medical cannabis dispensary.	Washington State, USA. 2007–2008, access points for medical cannabis dispensing in urban centers were informally tolerated.	37 chronically ill, qualified medical cannabis users. 41 (21–61) years. 65% men.	25% qualified with intractable pain. 51% used medical cannabis to reduce musculoskeletal pain.	59% of the participants reported that 3.4 grams of medical cannabis provided 97% pain relief for 65 h.	Scholarship funding *National Science Foundation Graduate Research Fellowship*
Alexandre 2011	To identify patient’s expectations and experience of the enrollment to the Rhode Island medical cannabis program. Qualitative: Semi-structured face-to-face interviews of patients enrolled in the medical cannabis program; recruitment via an information sheet distributed by the Rhode Island Patient Advocacy Coalition (RIPAC), supporting patients in the use of medical cannabis.	Rhode Island, USA. 2009–2010, legal MC use.	15 medical cannabis qualified users enrolled in the medical cannabis program. 23–60 years. 67% men.	Not reported for the study sample (67% of registered users diagnosed with chronic or debilitating disease or treatment, including chronic pain not related to cancer).	Reports of significant relief from pain.	No funding
Boehnke et al. 2016	To examine whether using medical cannabis for chronic pain changed individual patterns of opioid use. Quantitative: Retrospective cross-sectional survey (online questionnaire carried out in collaboration with an medical cannabis dispensary)	Michigan, USA. 2013–2015 Legal MC use.	185 qualified medical cannabis users who completed the 2011 Fibromyalgia Survey Criteria. 18–75 years. 64% men.	Chronic pain.	Medical cannabis use was associated with a 64% decrease in opioid use, decreased number and side effects of medications, and an improved quality of life (45%).	N/A
Bonn-Miller et al. 2014	To describe population. To examine association psychological & pain symptoms vs. medical cannabis use motives. Quantitative: Cross-sectional questionnaires; recruitment via an medical cannabis dispensary.	California, USA. Legal medical cannabis use.	217 qualified medical cannabis users receiving medical cannabis at dispensary. 41.2 ± 14.9 years. 73% men.	62% reported anxiety, 58% chronic pain, 49% stress, 48% insomnia, 45% depression, 30% appetite, 26% headaches, 22% nausea, 20% muscle spasms, 19% PTSD; less than 10% of the sample reported to use MC against cancer.	Regardless of condition, medical cannabis reported as moderately to mostly helpful.	(Mixed) Research grant *VA Clinical Science Research and Development (CSR&D) Career Development Award-2* Local resource funding *San Francisco Patient and Resource Center*
Bottorff et al. 2011	To describe perceived medical cannabis health effects. Qualitative: Semi-structured, individual face-to-face or telephone interviews; recruitment through an online forum and through compassion centers.	British Columbia, Canada. 2007–2008, Marihuana Medical Access Regulations * but adults recruited from tolerated but illegal dispensaries.	23 self-reporting medical cannabis users. 45 (25–66) years. 43% men.	26% HIV/AIDS 22% fibromyalgia 17% arthritis 13% mood/anxiety disorders.	Reports of immediate effects and, for the first time in many years, participants “could manage life again.”	N/A
Bruce et al. 2018	To learn more on how medical cannabis is used by persons living with chronic conditions in tandem with or instead of prescription medications. Qualitative: Semi-structured telephone interviews with open-ended questions; recruitment through flyers at medical cannabis dispensaries.	Illinois, USA. Legal medical cannabis use.	30 qualified medical cannabis users. 44.6 ± 15.9 years. 63% men.	23% rheumatoid arthritis 20% Crohn’s disease 20% spinal cord injury/disease 13% cancer 10% severe fibromyalgia.	Medical cannabis perceived as acting more quickly, having longer effects, reducing potential harm versus opioids/narcotics. Multiple benefits replacing a range of medications.	Fellowship grant *Provost’s Collaborative Research Fellowship, DePaul University*
Brunt et al. 2014	To assess therapeutic satisfaction with pharmaceutical-grade cannabis. To compare the subjective effects among the available strains. Quantitative: Questionnaires; recruitment through pharmacies specialized in medical cannabis distribution.	The Netherlands. 2011-2012, pharmaceutical-grade cannabis distributed for medicinal purposes since 2003.	113 qualified medical cannabis users. 52.8 ± 12.3 years. 49% men.	53% chronic pain 23% multiple sclerosis; only 11% indicated to use medical cannabis against cancer.	86% (almost) always experienced therapeutic satisfaction, mainly pain alleviation.	Governmental funding *Ministry of Health, Welfare and Sport*
Coomber et al. 2003	To report the experiences of medical cannabis users. Qualitative: Semi structured interviews; recruitment via advertisements in newspapers, disabled people’s organizations or friends.	UK. Illegal.	33 self-identified medical cannabis users. 44 (26–65) years. 58% men.	To relieve symptoms of chronic illness or disability: 42% multiple sclerosis 27% arthritic/rheumatoid complaints.	Medical cannabis perceived to be highly effective in treating symptoms, to complement existing medication, and to produce fewer unwanted effects.	N/A
Corroon et al. 2017	To survey cannabis users to determine whether they had intentionally substituted cannabis for prescription drugs. Online survey, recruitment through social media, cannabis dispensaries and word of mouth.	83% of the USA (all 50 states represented) and over 42 countries represented. 2013–2016 Legality differed between the USA and countries.	Convenience sample of 2 774 cannabis users. 63% were under 36 y, 56% men. 60% identified themselves as medical cannabis users.	1040/2774 (37%) of respondents reported pain and/or intractable pain.	46% have substituted cannabis for prescription drugs.	Research grant *NIH NCCAM K01ATTA (Ste-Marie et al.,*^2016^*)*
Cranford et al. 2016	To examine the prevalence and correlates of vaporization as a route of cannabis administration in medical cannabis users. Quantitative: Data from the screening assessment; recruitment at medical cannabis clinics.	Michigan, USA. 2014–2015 Legal medical cannabis use.	1485 adults seeking medical cannabis certification either for the first time or as a renewal (66%). 45.1 ± 13 years. 57% men.	91% severe chronic pain 26% severe and persistent muscle spasms.	not reported	Research grant *National Institute on Drug Abuse (NIDA), National Institutes of Health*
Crowell 2017	To ascertain the impact of medical cannabis on patients in New Jersey. Quantitative: Survey with open-ended questions; recruitment via a non-profit organization dispensing medical cannabis	New Jersey, USA. Legal medical cannabis use.	955 qualified medical cannabis users. 49.3 ± 13.6 (9–84) years. 51% men.	17 conditions were listed, including: 28% intractable skeletal spasticity 24% chronic/severe pain 16% multiple sclerosis 11% inflammatory bowel disease.	Improvement to general condition and quality of life. Decrease in pain, inflammation, nausea, intraocular pressure, spasms, seizure. Increase in appetite, mobility, mood and energy.	N/A
Degenhardt et al. 2015	To investigate patterns and correlates of cannabis use in people who had been prescribed opioids for chronic non-cancer pain. Qualitative: Interview; recruitment via a database of pharmacies and chemists across Australia.	Australia. Legal medical cannabis use.	242 patients prescribed opioids for chronic non-cancer pain which had used cannabis for pain. 48.7 ± 10.1 years. 62.5% men.	Chronic non-cancer pain, including: 84% back/neck problems 57% arthritis/rheumatism.	Among those using cannabis for pain, the average pain relief was 70% while the average pain relief from prescribed opioids was 50%.	Research grant *Australian National Health and Medical Research Council*
Erkens et al. 2005	To characterize medical cannabis users, symptoms and conditions; daily use of medical cannabis. Quantitative: Structured questionnaire; recruitment via pharmacies.	Netherlands. 2003–2004, since 2003, pharmaceutical-grade cannabis is distributed for medicinal purposes.	200 patients who filled a prescription for medical cannabis. ≥ 30 years. 33% men.	Cannabis mainly used for chronic pain (including rheumatic disease) and muscle cramp/stiffness.	Not reported	Governmental funding *Ministry of Health, Welfare and Sports, The Netherlands*
Fanelli et al. 2017	To present the first snapshot of the Italian experience with cannabis use for chronic pain over the initial year of its use. Quantitative: Retrospective case series (physician-filled case report form); recruitment via second-level pain clinics.	Pisa, Italy. 2015–2016, initial year of authorized medical cannabis use for chronic pain. Legal medical cannabis use.	614 qualified medical cannabis users. 61.3 ± 15.3 years. 29% men.	91% chronic pain.	49% reported an improvement associated with the therapy. 15% stopped the treatment due to side effects (none of which were severe).	N/A
Gorter et al. 2005	To investigate indications for cannabis prescription. To assess cannabis efficacy and side effects. Quantitative: Standardized questionnaire; recruitment via questionnaires accompanying shipment of medical-grade cannabis directed to both patient and prescribing physician.	Netherlands. 1997–1999, before legalization but consumption of small amounts under certain conditions was then condoned.	107 patients receiving medical-grade cannabis on prescription. Median 58 years. 45% men.	39% neurologic disorders 21% musculoskeletal/connective tissue disorders 14% malignant tumors and symptoms thereof.	64% reported good to excellent effect on their symptoms. Generally mild side effects.	Non-governmental organization funding *Maripharm*
Grella et al. 2014	To collect descriptive data on individuals using medical cannabis dispensaries. Mixed Focus groups and survey; recruitment via medical cannabis dispensaries. S	California, USA. May–October 2012, legal medical cannabis use.	Users of medical cannabis dispensaries: Focus groups: *n* = 30, 38 ± 12 (20–64) y, 70% men. Survey: *n* = 182, 28.4 ± 5.3 y, 74% men.	Conditions most often cited (not mutually exclusive): 60% anxiety 56% insomnia*/*sleep problems 33% depression 42% chronic (non-cancer) pain.	Nearly all believed MC beneficial in treating their health problems.	Governmental funding *Los Angeles County Department of Public Health, Substance Abuse Prevention and Control Programs*
Groten-hermen & Schnelle 2003	To investigate indications for cannabis prescription. To assess cannabis efficacy and side effects. Quantitative: Questionnaires; recruitment via an medical cannabis association.	German speech area of Europe. 2001: illegal use of natural cannabis products but THC could be prescribed.	143 participants with cannabis or THC experience. Median 40.3 (16–87) years. 61% men.	28% neurological symptoms 25% painful conditions.	75% reported their conditions much improved by cannabis or THC. 73% reported no side effects.	N\A
Haroutounian et al. 2016	To determine the long-term effect of medical cannabis on pain and functional outcomes in participants with treatment resistant chronic pain. Quantitative: Prospective, open-label, single-arm longitudinal study (questionnaires); recruitment via an ambulatory pain clinic.	Jerusalem, Israel. 2010–2013, legal medical cannabis use.	206 qualified medical cannabis users. 51.2 ± 15.4 years 62% men.	93% chronic non-cancer pain, including: 37% musculoskeletal pain 34% peripheral neuropathic pain 19% radicular low back pain.	Pain symptom score improved (*P* < 0.001) in association with improvement in physical function (*P* < 0.001). 9 (4%) discontinued treatment due to mild to moderate AEs; 2 (1%) discontinued to serious side effects (1 elevated liver transaminases, 1 elderly admitted to an Emergency Department in a confusional state).	Research grant Support from the Hadassah-Hebrew University Pain Relief Unit
Harris et al. 2000	To better understand relationships between past experience with drugs and reasons for cannabis use; perceived effectiveness of cannabis as a therapeutic agent. Quantitative: Questionnaires; recruitment via advertisements posted at the Cannabis Cultivator’s Club.	California, USA (after 1996) Legal MC use.	100 Cannabis Cultivator’s Club members. 40 ± 8 years. 78% men.	33% AIDS (appetite) 21% musculoskeletal/arthritis 15% gastrointestinal (most often nausea) 15% psychiatric (primarily depression) 13% neurologic and non-musculoskeletal pain syndromes.	66% rated effectiveness as 80% compared with 52% for other medications. 56% reported no side effects. Less severe side effects than other treatments. Anxiety effects frequently reported on the checklist but not listed as side effects.	Research grant *US Public Health Service grants, National Institute on Drug Abuse*
Hazekamp & Heerdink, 2013	To analyze the incidence and prevalence of medical cannabis use and characteristics of users. Quantitative: Retrospective database study; recruitment through the Dutch Foundation for Pharmaceutical Statistics and the only Dutch pharmacy specialized in medical cannabis dispensing.	Netherlands. 2003–2010, pharmaceutical-grade cannabis distributed for medicinal purposes since 2003.	5540 patients with ≥ 1 medical cannabis prescription. 56 (14–93) years. 43% men.	Reason for medical cannabis use not reported but 43% had analgesics prescribed in the 6-month period preceding start of MC use. Only 2.7% received oncologicals, thus cancer is unlikely to be present in all pain patients in the study.	not reported	N/A
Hazekamp et al. 2013	To compare different administration forms of cannabinoids and identify their relative advantages and disadvantages as described by actual users. International, web-based, cross-sectional survey; recruitment via the official website of the International Association for Cannabinoid Medicines.	31 countries including the USA (40 states represented), Germany, France, Canada, Netherlands & Spain. 2009–2010, legality differed by country.	953 adults self-reporting experience with ≥ 2 different cannabinoid-based medicines or administration forms, 87% current medical cannabis users. 40.7 (14–76) years. 64% men.	Top 5 conditions: 12% back pain 7% sleeping disorder 7% depression 6% pain resulting from injury or accident 4% multiple sclerosis. Pain medication was consumed by 53.6% of medical cannabis users	Herbal medical cannabis received higher appreciation than pharmaceutical cannabinoids. Side effects: irritation of the lungs (inhalation), drowsiness, uncontrollable appetite, “getting high”.	Non-governmental organization funding *Dutch Association for Legal Cannabis and its Constituents as Medicine (NCSM foundation)*
Hoffman et al. 2017	To begin the development of a cannabis use registry in Oregon. Qualitative: Semi-structured interviews; recruitment via an outpatient healthcare clinic.	Oregon, USA. July–August 2015: legal medical cannabis use, nonmedical used became legal on July first.	22 qualified medical cannabis users. Median 38 (20–64) years. 45% men.	59% musculoskeletal pain 27% PTSD.	Some reported physiologic relief from pain, others said it helped take their mind off of it. Respondents felt that the benefits outweighed the risks.	Research grant *National Institute of Drug Abuse supported this study*
Ilgen et al. 2013	To describe adults seeking medical cannabis; To compare them with those renewing their medical cannabis card on substance use; pain; functioning. Quantitative: Questionnaires; recruitment at the waiting room of an medical cannabis clinic.	Michigan, USA. Legal medical cannabis use.	348 adults seeking medical cannabis certification either for the first time (56%) or as a renewal (44%). 41.5 ± 12.6 years. 66% men.	87% used medical cannabis for pain relief, including 7% for musculoskeletal problems.	Not reported	N/A
Kilcher et al. 2017	To study medical uses of cannabinoids as part of the Swiss Federal Office of Public Health (FOPH) programme of exceptional licenses. Quantitative: Data from the formal requests for medical use of cannabinoids; recruitment via formal requests of medical cannabis use.	Switzerland. 2013–2014, exceptional licenses for medical use of cannabinoids.	1193 qualified medical cannabis users. 57 ± 15 years. 43% men.	Most common symptoms:49% chronic pain40% Spasticity Diagnosis:25% musculoskeletal conditions22% multiple sclerosis.	Licences were initially granted for 6 months, physicians requested extensions when the treatment had been satisfactory. The number of extensions increased from 26% in 2013 to 39% in 2014.	N/A
Lavie-Ajayi & Shvartzman 2018	To evaluate the subjective experience of pain relief by medical cannabis. Qualitative: In-depth semistructured interviews; recruitment through a pain clinic.	Israel. 2016–2017, legal medical cannabis use.	19 patients seeking treatment with medical cannabis. 52 (28–79) years. 53% men	Chronic pain:37% arthritis32% spinal cord injuries32% CRPS 5% cancer.	Immediate sensation of chronic pain relief, improved sleep quality, improved life quality. Side effects: increased appetite (74%), drowsiness (67.1%), ocular irritation (40.7%), lack of energy (37.5%), memory impairment (31.6%), palpitations (15.4%), and paranoia (15.2%) or confusion (12.4%).	Research grant *Ben Gurion University of the Negev, Faculty of Humanities and Social Sciences.*
Lintzeris et al., 2018	To explore patterns of medical cannabis use. Quantitative: Online survey; recruitment trough online media, consumer group webpages, and medical cannabis consumer forums.	Australia. 2016, illegal medical cannabis use.	1748 medical cannabis users. 37.9 years. 68% men.	51% anxiety, 50% back pain, 49% depression, 44% sleep problems, 26% neck pain, 23% PTSD. 69.4% of respondents used medical cannabis to manage pain.	Most participants reported that medical cannabis reduced significantly chronic pain. Side effects: increased appetite (74%), drowsiness (67%), ocular irritation (41%), lack of energy (38%), memory impairment (32%), palpitations (16%), paranoia (15%) or confusion (12%).	(Mixed) Research grant *Australian Research Council and the National Health and Medical Research council (NHMRC)* Local research grant *Lambert Initiative for Cannabinoid Therapeutics*
Lucas & Walsh 2017	To describe medical cannabis access, use and substitution for patients enrolled in the Canadian Marihuana for Medical Purposes regulations. Quantitative: Online cross-sectional survey; recruitment through a licensed producer of cannabis.	Canada. July 2015, legal medical cannabis use (Marihuana for Medical Purposes Regulations *).	271 qualified medical cannabis users (Marihuana for Medical Purposes Regulations). 40 (20–77) years. 73% men.	53% pain-related conditions: 36% chronic pain, 12% arthritis, 5% headache. Most highly endorsed symptoms: 73% chronic pain, 60%, stress, 57% insomnia, 46% depression, 32% headache.	95% reported that cannabis often or always helped alleviate their symptoms.	Research grant *Institute for Healthy Living and Chronic Disease*
Lynch et al. 2006	To describe medical cannabis users. Quantitative: Structured follow-up questionnaire; recruitment of patients followed at a tertiary care pain management center.	Nova Scotia, Canada. 2001-2005, legal medical cannabis use (Marihuana Medical Access Regulations Marihuana Medical Access Regulations *).	30 qualified medical cannabis users (Marihuana Medical Access Regulations). 45 (31–61) years. 60% men.	Chronic severe pain that had not responded to traditional approaches: 47% neuropathic pain 13% low back pain 10% arthritis.	93% reported moderate or greater pain relief. 95% reported subjective improvement in function. No serious adverse events reported.	N/A
Nunberg et al. 2011 and Reinarman et al. 2011	To describe medical cannabis users: demographics; symptoms; physician evaluations; conventional treatments tried; use practices. Quantitative: Physician records and patients’ questionnaire; recruitment through nine medical cannabis clinics.	California, USA. June–August 2006, legal medical cannabis use.	1746 medical cannabis applicants. 33% ≥ 45 years. 75% men.	82.6% report using medical cannabis to relieve pain. 58.2% diagnosed with chronic pain disorders, including: 26% low back pain 18% arthritis 2% fibromyalgia.	Patients typically report at least one therapeutic benefit: 83% relief of pain 41% muscle spasms 41% headache 38% anxiety 28% nausea and vomiting 26% depression.	(Mixed funding) Research grant *RAND Corporation;* Non-governmental organization funding Cannabis “industry” *MediCann;* Private Foundation *Rosenbaum Foundation*
Ogborne et al. 2000	To explore reasons for medical cannabis use; medical cannabis effects; methods and patterns of use; experiences with physicians; encounters with the law. Qualitative: Interview; recruitment through advertisements in newspapers and on bulletin boards at an Addiction Research Foundation and at different town locations (bookstores, grocery stores, restaurants, laundromats, etc).	Toronto, Canada. Before the 2001 *Marihuana Medical Access Program*.	50 self-identified medical cannabis users. 38 (26–57) years. 66% men.	22% HIV/AIDS-related symptoms 14% chronic/recurrent pain due to injury of unknown origin 12% depression 2% arthritis.	medical cannabis described as superior to other treatments. Reported lethargy, apathy, cough or throat irritation from smoking, thirst, loss of concentration, short-term memory loss, paranoia, and depression.	N/A
Pedersen & Sandberg 2013	To investigate the medical motives of Norwegian cannabis users. Qualitative: Semi-structured interviews; recruitment through internet advertisements, authors‘ own social networks, among students at the University of Oslo, and from organizations such as the National Organization for the Reform of Marijuana Laws.	Norway. 2006–2010, illegal.	100 long-term cannabis users (25 stated explicitly they used cannabis medically). 20–62 years. 88% men.	Cannabis was used therapeutically for conditions such as multiple sclerosis, attention deficit hyperactivity disorder and rheumatism, as well as for quality of life conditions such as quality of sleep, relaxation and wellbeing.	Cannabis typically described as useful for treating stress, insomnia and pain, as well as for relaxation.	Research grant *Research Council of Norway*
Perron et al. 2015	To better elucidate, among MC users with and without concurrent use of prescription pain medication (PPM): patterns of alcohol and other drug use; functioning; perceived efficacy of pain treatments. Quantitative: Questionnaires; recruitment via a survey conducted among persons seeking medical cannabis certification or recertification at an medical cannabis certification clinic.	Michigan, USA. Legal medical cannabis use.	273 adults reporting past-month cannabis use for pain-related purposes (subsample of Ilgen et al.’s 2013 study). 40.3 ± 12.5 years. 69% men.	Subset of subjects who endorsed using cannabis in the past month specifically for pain reduction.	Prescription pain medication (PPM) users perceived cannabis as more efficacious than PPMs.	Research grant *National Institute on Drug Abuse grant*
Piper et al. 2017	To provide an in-depth qualitative exploration of patient perspectives on the strengths and limitations of medical cannabis. Online survey with open-ended questions; recruitment via medical cannabis dispensaries.	Maine, Vermont, and Rhode Island, USA. 2015–2016 (chronic pain was not a condition to become part of the Vermont registry).	984 members of medical cannabis dispensaries. 49.1 ± 0.5 years. 47% men.	64% reported a diagnosis of chronic pain:91% back/neck pain30% neuropathic pain23% postsurgical pain22% abdominal pain20% chronic pain after trauma/injury.	75% relief of symptoms. Reported benefits: pain relief, better sleep, safe/natural (limited addictive potential), quality of life, functionality. Negative themes: respiratory effects, increased appetite, cognitive (decrease ability to concentrate, non-alert feeling…).	(Mixed) Nonprofit organization funding *Center for Wellness Leadership* Local resource funding *Wellness Connection of Maine (Burstein,*^2015^*);* Research grant *National Institute of Drug Abuse*
Reiman 2009	To examine drug and alcohol use, and the occurrence of substitution among medical cannabis users. Quantitative: Survey data collected at a medical cannabis dispensing collective; recruitment through an medical cannabis dispensing collective.	California, USA. Legal medical cannabis use.	350 medical cannabis users 39 (18–81) years. 68% men.	52% use cannabis for a pain related condition, including 45% who used it against pain resulting from an alcohol related accident. 75% use cannabis for a mental health issue.	65% use medical cannabis as a substitute for alcohol, illicit or licit drugs with less adverse side effects.	N/A
Reiman et al. 2017	To gather the impressions of patients who have used cannabis on how it compares with pain medications. Quantitative: Cross-sectional survey; recruitment through e-mails addressed to medical cannabis patients of an medical cannabis patient database (67,422 patients).	California, USA.	2897 medical cannabis respondents seeking medical cannabis certification. ≥ 20 years. 55% men.	63% pain-related conditions including back pain and arthritis.	Respondents overwhelmingly reported that cannabis provided relief on par with their other medications, but without the unwanted side effects.	N/A
Sagy et al. 2019	To investigate the characteristics, safety and effectiveness of medical cannabis in fibromyalgia over a period of 6 months. Quantitative: Questionnaire; recruitment via medical cannabis provider.	Israel. 2015–2017, legal medical cannabis use.	367 fibromyalgia patients, qualified medical cannabis users. 52.9 (± 15.1) years. 18% men.	100% fibromyalgia.	Overall pain intensity assessed by NRS reduced from a median of 9.0 at baseline to 5.0 after 6 months of medical cannabis treatment (*P* < 0.001). Side effects: dizziness (7.9%), dry mouth (6.7%), nausea/vomiting (5.4%), hyperactivity (5.5%), increased appetite (3.8%).	N/A
Schnelle et al. 1999	Quantitative: questionnaire; recruitment via an medical cannabis association.	Germany, Austria and Switzerland. 1998-1999.	128 qualified medical cannabis users. 37.5 ± 9.6 y 68% men	12% depression 11% multiple sclerosis 9% HIV infection 5% back pain.	Symptoms improvement from much (72.2%), to none (4.8%). 1.6% experienced worsening of symptoms. 70.8% experiences no adverse effects.	N/A
Sexton et al. 2016	To collect epidemiologic data to inform medical practice, research, and policy to provoke discussion about the discrepancies between medico-legal recommendations and patient-reported outcomes. Quantitative: Cross-sectional online survey); recruitment through links posted on University (Bastyr University California (US)) websites, social media and cannabis dispensaries.	Respondents came from 18 countries, with the USA (78%), the UK (6%), and Canada (3%) being the most represented. 2013–2016. Legality varies across countries.	Convenience sample of 1429 self-identified medical cannabis users. 36.3 ± 14 (15-80) years. 55% men.	61% pain 58% anxiety 50% depression 35.5% headache/migraine 27% nausea 18% muscle spasticity 17% arthritis 15% irritable bowel 11.5% intractable pain.	On average, participants reported an 86% reduction in symptoms.	Research grant *NIH NCCAM K01ATTA (Ste-Marie et al.,*^2016^*)*
Shah et al. 2017	To examine clinical and treatment characteristics for patients who are admitted to a 3-week outpatient inter-disciplinary chronic pain rehabilitation program. Quantitative: Self-report questionnaire and chart review; recruitment of patients admitted to a 3-week outpatient inter-disciplinary chronic pain rehabilitation program.	The USA. March–December 2015. Not reported	24 patients with THC positive urine test participating to a pain rehabilitation program. 45.4 ± 15.3 years. 42% men.	Chronic pain.	Not reported	N/A
Shiplo et al. 2016	To examine modes of medical cannabis delivery following regulatory changes in 2014. Quantitative: Online cross-sectional survey; recruitment via nine Health Canada licenced medical cannabis producers.	Canada. April–June 2015. Legal medical cannabis use.	Convenience sample of 364 qualified medical cannabis users. 40.8 ± 12.6 years. 58% men.	45% for pain relief (chronic pain and fibromyalgia) 15% mental health 10% central nervous system.	not reported	Research grant *Canadian Institute of Health Research (CIHR) Training Grant Program in Population Intervention for Chronic Disease Prevention*
Ste-Marie et al. 2012	To document the self-identified prevalence of cannabinoid use in fibromyalgia patients seen in a fibromyalgia clinic. Qualitative: Retrospective chart review; recruitment via a tertiary care pain center.	Montreal, Canada. 2005–2010. Legal medical cannabis use.	59 medical cannabis users with a diagnosis of fibromyalgia. 24% used prescription cannabinoids. 45 ± 10 y 33% men.	Fibromyalgia (61%) or regional pain syndrome and spinal pain, rheumatic disease, neurologic condition.	Not reported	Research grant *Louise and Alan Edwards Foundation*
Ste-Marie et al. 2016	To examine the prevalence of cannabis use among rheumatology patients. To compare the clinical characteristics of medical cannabis users and nonusers. Quantitative: Cross-sectional survey (questionnaires); recruitment via an university-affiliated community rheumatology clinic.	Ontario, Canada. April–May 2014. Legal medical cannabis use.	28 current medical cannabis users. 52.7 ±13.6 years. 43% men. 15 previous medical cannabis users, 62.8 ± 14.4 y, 26% men.	Specific rheumatic disease : 54% osteoarthritis or spinal pain 32% inflammatory arthritis 18% fibromyalgia.	Medical cannabis reported to relieve pain, anxiety, nausea, improve sleep and appetite.	Research grant *Louise and Alan Edwards Foundation*
Swift et al. 2005	To learn more about: patterns of use; experiences and concerns; interest in participating in a medical cannabis trial. Quantitative: Mailed questionnaires; recruitment through opportunistic media stories in newspapers, on radio and television.	Australia. 2003-2004. Illegal.	128 medical cannabis users Median 45 (24–88) years. 63% men.	Condition: 60% depression 53% chronic pain 38% arthritis.	86% reported great relief from cannabis. Typically perceived as superior to other medications in terms of undesirable effects, and the extent of relief provided. 15% had stopped, 16% disliked the side effects or route of use (each 3/19).	N/A
Troutt & DiDonato, 2015	To examine medical cannabis users: characteristics; perceptions; behaviors. To learn about experiences with cannabis before legalization. Quantitative: Anonymous online survey; recruitment: via four medical cannabis dispensaries.	Arizona, USA. After the 2012 Arizona Department of Health Services Medical Marijuana Rules.	367 patients recruited from medical cannabis dispensaries. 45.78 ± 13.76 (18–83) years. 64% men.	87% chronic pain 24.5% arthritis 11% osteoarthritis 7% fibromyalgia.	70% experienced a lot of or almost complete relief.	N/A
Walsh et al. 2013 and Belle-Isle et al. 2014	To examine: cannabis use history; medical conditions and symptoms; patterns of use; modes of access; perceived effectiveness. Quantitative: Survey (online or at a cannabis dispensary); recruitment through local medical cannabis dispensaries and national organizations that assist medical cannabis users.	British Columbia, Canada. 2011–2012. Legal medical cannabis use.	628 self-identified current medical cannabis users. 39.1 ± 13.1 years. 71% men.	Pain, including chronic, spinal and non-spinal pain, arthritis (82%), anxiety, and sleep problems.	Cannabis perceived to provide effective symptoms relief: 72% reported medical cannabis always helpful, 24% often helpful.	Research grant *UBC Institute for Healthy Living and Chronic Disease Prevention*
Ware et al. 2003	To determine current prevalence of medical cannabis in chronic non-cancer pain; estimate the dose size and frequency of cannabis use; describe main symptoms for which relief was sought. Quantitative: Cross-sectional survey; recruitment of all patients entering the ambulatory pain management unit of the Queen Elizabeth II Health Sciences Center.	Nova Scotia, Canada. June to July 2001. Legal medical cannabis use.	09 chronic non-cancer pain patients.35% had ever used cannabis, 15% have used cannabis for pain relief, and 10% were current MC users for pain relief.	Of MC users: 50% trauma/surgery 6% arthritis 6% multiple sclerosis.	Improved pain, sleep and mood. 78% of medical cannabis users reported at least moderate relief of pain. 25% reported no side effects, 37% very mild, 28% moderate, 9% strong side effects, no severe side effects.	(Mixed) University funding *Faculty of Medicine *Department of Anesthesia; Non-governmental organization funding *Research-based pharmaceutical companies*
Webb & Webb 2014	To discover the benefits and adverse effects perceived by medical cannabis users, especially with regards to chronic pain. Quantitative: Survey (questionnaires); recruitment via questionnaires hand-delivered to medical cannabis certified patients re-applying for certification.	Hawaii, USA. 2010–2011. Legal MC use.	94 patients re-applying for medical cannabis certification. 49.3 years.	97% used cannabis primarily for chronic pain.	64% relative decrease in average pain. 71% reported no adverse effects, 6% reported a cough or throat irritation.	N/A
Zaller et al. 2015	To characterize socio-demographics and reasons for medical cannabis use among dispensary patients. Quantitative: Cross-sectional survey (questionnaires); recruitment through Compassion Centers of the Department of Health.	Rhode Island, USA. After the 2013 authorization for medical cannabis dispensaries.	200 qualified medical cannabis users. Median 41 (18–76) years. 73% men.	The most common reason for medical cannabis use was chronic pain management.	Most participants report that medical cannabis improves their pain symptomology. 91.5% report less unwanted side effects than with prescription medications.	N/A

1 In Canada, 1999: right to possess cannabis for medical purposes (MC); 2001: Marihuana Medical Access Regulations (MMAR) enabled individuals with the authorization of their health care practitioner to access dried MC by producing their own plants, designating someone to produce for them or purchasing Health Canada supply; 2013: Marihuana for Medical Purposes Regulations (MMPR) commercial production and distribution of MC; 2015: production and sale of cannabis oil, fresh buds and leaves; 2016: Access to Cannabis for Medical Purposes Regulations (ACMPR) set out provisions for individuals to produce a limited amount for their own medical purposes (https://www.canada.ca/en/health-canada/services/publications/drugs-health-products/understanding-new-access-to-cannabis-for-medical-purposes-regulations.html).

2 As of May 18, 2021 36 states and 4 territories of the United States of America allow for the medical use of cannabis products (https://www.ncsl.org/research/health/state-medical-marijuana-laws.aspx).

3 Abbreviations: *AE*: adverse effects; *ED*: emergency department; *MC*: medical cannabis/cannabis for therapeutic purpose/medical marijuana; *MMAR*: Marihuana Medical Access Regulations; *MMPR*: Marihuana for Medical Purposes Regulations; *NR*: not reported; *PTSD*: Post-traumatic stress disorder; *THC*: delta-9-tetrahydrocannabinol; NRS: numeric rating scale; *CRPS*: complex regional pain syndrome

### Participants’ characteristics

Participants’ characteristics are described for each study in Table 1 and summarized in Table 4.

### Patterns of MC use

Reported patterns of MC use for each study are presented in Table 2 and user experiences relating to the pattern or mode of use are shown in Table 3. The mode of cannabis administration was described in 36 studies. The most common form of MC consumption was inhalation (reported in 35 studies), either via smoking (joint or blunt, joint with tobacco, pipe, water pipe) or vaping (vaporizer) (Swift et al., 2005; Aggarwal et al., 2009; Hazekamp et al., 2013; Ste-Marie et al., 2016; Bottorff et al., 2011; Bruce et al., 2018; Coomber et al., 2003; Erkens et al., 2005; Haroutounian et al., 2016; Harris et al., 2000; Hoffman et al., 2017; Lucas & Walsh, 2017; Lynch et al., 2006; Ogborne et al., 2000; Piper et al., 2017; Reinarman et al., 2011; Schnelle et al., 1999; Sexton et al., 2016; Shiplo et al., 2016; Ste-Marie et al., 2012; Troutt & DiDonato, 2015; Walsh et al., 2013; Ware et al., 2003; Brunt et al., 2014; Cranford et al., 2016; Crowell, 2017; Fanelli et al., 2017; Grella et al., 2014; Grotenhermen & Schnelle, 2003; Shah et al., 2017; Zaller et al., 2015; Lavie-Ajayi & Shvartzman, 2018; Lintzeris et al., 2018; Reiman, 2007; Sagy et al., 2019). Reported smoking prevalence ranged from 20 (Erkens et al., 2005) to 91% (Cranford et al., 2016) and vaping prevalence from 7 (Crowell, 2017) to 53% (Shiplo et al., 2016). Ingested (cannabis tea, baked goods, oils, tinctures, tablets and capsules) (Hazekamp et al., 2013; Ste-Marie et al., 2016; Bruce et al., 2018; Erkens et al., 2005; Haroutounian et al., 2016; Hoffman et al., 2017; Lucas & Walsh, 2017; Lynch et al., 2006; Piper et al., 2017; Reinarman et al., 2011; Sexton et al., 2016; Troutt & DiDonato, 2015; Walsh et al., 2013; Ware et al., 2003; Brunt et al., 2014; Cranford et al., 2016; Crowell, 2017; Fanelli et al., 2017; Grella et al., 2014; Grotenhermen & Schnelle, 2003; Reiman et al., 2017; Shah et al., 2017; Zaller et al., 2015; Sagy et al., 2019) and topical administration (Ste-Marie et al., 2016; Bruce et al., 2018; Hoffman et al., 2017; Lucas & Walsh, 2017; Sexton et al., 2016; Cranford et al., 2016) were less common forms of MC use (reported in 25 and 6 studies, respectively). The reported prevalence of ingested MC varied from 0.5 (Sexton et al., 2016) to 70% (Erkens et al., 2005) and the prevalence of topical administration varied from 0.6 (Sexton et al., 2016) to 11% (Cranford et al., 2016). A combined mode of cannabis consumption (e.g., both smoked MC and edible MC products) was also reported (Haroutounian et al., 2016; Shiplo et al., 2016; Ste-Marie et al., 2012; Grotenhermen & Schnelle, 2003). Frequency and quantity of MC consumption was described in 23 (Swift et al., 2005; Aggarwal et al., 2009; Hazekamp et al., 2013; Coomber et al., 2003; Erkens et al., 2005; Harris et al., 2000; Lucas & Walsh, 2017; Lynch et al., 2006; Ogborne et al., 2000; Reinarman et al., 2011; Sexton et al., 2016; Shiplo et al., 2016; Troutt & DiDonato, 2015; Walsh et al., 2013; Ware et al., 2003; Bonn-Miller et al., 2014; Brunt et al., 2014; Cranford et al., 2016; Crowell, 2017; Grella et al., 2014; Shah et al., 2017; Zaller et al., 2015; Lintzeris et al., 2018) and 22 studies (Aggarwal et al., 2009; Hazekamp et al., 2013; Ste-Marie et al., 2016; Haroutounian et al., 2016; Harris et al., 2000; Lucas & Walsh, 2017; Lynch et al., 2006; Nunberg et al., 2011; Ogborne et al., 2000; Reinarman et al., 2011; Sexton et al., 2016; Shiplo et al., 2016; Ste-Marie et al., 2012; Troutt & DiDonato, 2015; Walsh et al., 2013; Bonn-Miller et al., 2014; Brunt et al., 2014; Cranford et al., 2016; Fanelli et al., 2017; Grotenhermen & Schnelle, 2003; Zaller et al., 2015; Lavie-Ajayi & Shvartzman, 2018; Sagy et al., 2019), respectively. Between 38 (Ware et al., 2003) and 90% (Brunt et al., 2014) of participants reported daily MC consumption. Consumed quantity of MC varied from 0.05 (Fanelli et al., 2017) to 1 gram per day (Harris et al., 2000).

**Table 2:** Patterns of medical cannabis use and utilization of medical cannabis as a substitute for prescription medications

Article	Mode of cannabis administration	Mode advantages	Mode Disadvantages	Quantity^**1**^/Frequency of cannabis use	Cannabis used as a substitute for prescription medications
Aggarwal et al. 2009	When mentioned, mainly smoking.	Not reported	Not reported	From “as needed” to over 10 times daily. From ½ to 14 g/week.	Not reported
Boehnke et al. 2016	Not reported	Not reported	Not reported	Not reported	45% of respondents reported a 64% reduction in opioid use with medical cannabis use. Decrease in the number of medications classes used with medical cannabis use (2.38 to 1.81, *P* < .001).
Bonn-Miller et al. 2014	Not reported	Not reported	Not reported	Participants used 2 to 3 times/day. They used 6–12 g/week.	Not reported
Bottorff et al. 2011	Primarily smoking.	Smoking: • convenient • affordable • more effective regulation of dosing.	Smoking-related concerns: • coughing • breathing difficulties • fear of lung cancer.	Not reported	Not reported
Bruce et al. 2018	60% of participants preferred smoking; 20% vaporizing; 17% ingestion; 3% topical use.	Not reported	Not reported	Not reported	medical cannabis use reported as: • alternative to prescription (opioids, anticonvulsants, anti-inflammatories) or OTC medications; • complementary, with prescription medications; • a means for tapering off prescription medications.
Brunt et al. 2014	81% inhalation; 19% tea.	Not reported	Not reported	90% of participants used daily. Mean cumulative dose: 0.65 ± 0.63 g/day *[4.5 g/week]*.	Not reported
Coomber et al. 2003	73% smoking.	Smoking: less amount required than eating or drinking.	Not reported	48% used daily; 24% used 1–3 times/week. 24% used 1–3 joints/day.	Not reported
Corroon et al. 2017	Not reported	Not reported	Not reported	Not reported	Odds of reporting substitution 4.59 (95% CI, 3.87–5.43) times greater among self-identified medical cannabis than among non-medical cannabis users. Most common classes of drugs substituted: narcotics/opioids (36%), anxiolytics/BZD (14%), and antidepressants (13%).
Cranford et al. 2016	91% reported smoking; 44% eating, drinking, or ingesting; 39% vaping; 11% topical use. > 50% indicated > 1 mode for past month cannabis use.	Not reported	Not reported	74% of participants used almost daily. From none to > 1 ounce (14.5% of participants)/month *[0 to 6.5 g/week]*.	Not reported
Crowell 2017	Most frequent mode: 80% smoking; 7% vaporizing; 12% edibles.	Not reported	Not reported	3–4 times/day (41.6–37.9%); 1–2 times/day (38.7–27.1%).	At first visit: 50% of participants had reduced use of pain medication; at visit 2: 62.4%; at visit 3: 60%.
Erkens et al. 2005	70% as tea; 20% smoked.	Not reported	Not reported	1 to 4 times/day.	Not reported
Fanelli et al. 2017	Primarily cannabis tea (smoking cannabis not permitted in Italy). 92% used 22% THC/< 1% CBD Bedrocan.	Not reported	Not reported	From 56.7 ± 45.5 mg/day *[0.4 g/week]* at treatment initiation; to 67.0 ± 58.8 mg/day *[0.5 g/week]* at follow-up (98 ± 145 days).	Not reported
Grella et al. 2014	51% used a pipe*/*water pipe, 47% smoked joints or blunts; 23% used vaporizers; 16.5% edibles; 3.3% oral tincture.	Not reported	Not reported	2.5 ± 2.6 dispensary visits/month. 57% of focus group participants used several times daily.	A common theme among participants was the preference for using medical cannabis instead of prescription medications. In the previous 30 days, 7% had non-medical use of painkillers, 4% of stimulants, and 8% of tranquillizers.
Grotenhermen & Schnelle 2003	56% inhalation; 17% oral use; 23% used both modes.	Not reported	Not reported	Average doses of natural cannabis products (109 participants): 1.3 ± 0.9 (0.02–3.5) g/day *[9.1 g/week]*.	Not reported
Haroutounian et al. 2016	77% received cannabis cigarettes; 5% received a combination of cigarettes and drops; 10% only drops; 5% only cookies; 3% combination of cookies and drops.	Not reported	Not reported	Monthly prescribed cannabis: 43.2 ± 17.9 g/month	44% of participants on opioid therapy at baseline had discontinued (*P* < 0.001).
Harris et al. 2000	Mainly smoking.	Not reported	Not reported	65% daily use. 86% used ≥ 2 cigarettes/day. 1 g/day.	Not reported
Hazekamp et al. 2013	63% preferred smoking; 24% vaporizing; 8% food/tincture; 2.4% tea. Fewer participants had experience with dronabinol 11.3%, nabilone 2.1%, nabiximols 1.1%.	Not reported	Not reported	On average Times per day: smoking 6.0, vaporizing 5.2, tea 1.9, food/tincture 1.8. Grams per day: smoking 3, vaporizing 3, tea 2.4, food/tincture 3.4 g.	Not reported
Hoffman et al. 2017	73% smoking; 32% ingestion; 23% vaporizing; 9% topical use.	• Most felt vaporizing healthier than smoking. • Of those who ingested, most felt it more effective for pain relief than smoking.	Not reported	Not reported	Not reported
Lavie-Ajayi and Shvartzman 2018	Smoking and others (not reported)	Not reported	Unpleasant taste or smell of cannabis.	20–60 g/month	Reduction in side effects of prescription medication. Medical cannabis use reported as alternative to other medication used for sleeplessness, irritability, restlessness, inability to focus, and depression.
Lintzeris et al. 2018	Inhalation (83.4%)	Not reported	Not reported	Participants used 3 times/day	Not reported
Lucas and Walsh 2017	90% had tried joints, 86% vaporizers, 76% oral/edibles, 16% topical. Primary methods of use: 38% vaporizing, 25% smoking joints, 14% oral/edibles, 12% waterpipe/bongs, 11% pipes, 1% topicals. Preferred method: 44% vaporization, 23% edibles.	Not reported	Not reported	88% of participants used at least daily. Modal: 1–2 g/day *[7-14 g/week]*, with 29% (n = 79) using a larger amount.	63% of participants reported substitution for prescription medication. The most common form of substitution was for opioids (32%), BZD (16%), and antidepressants (12%).
Lynch et al. 2006	All participants reported smoking some of the time. 30% used both the smoking and oral routes; 7% used primarily the oral route.	Not reported	Not reported	1 to > 6 times/day. 2.5 g/day [17.5g/week].	70% decreased use of other medications that had been causing side effects (NSAIDs, opioids, and antidepressants).
Nunberg et al. 2011 & Reinarman et al. 2011	Not reported	Not reported	Not reported	Not reported	51% reported using cannabis as a substitute for prescription medications.
Ogborne et al. 2000	Mainly smoking.	Smoking: • enjoyable • immediate, effective • less expensive Eating/drinking: • “less of a head stone…” • longer lasting • no smell	Smoking: • Respiratory side effects (cough, throat irritation) Eating/drinking: • too slow • less effective • more difficult to regulate in terms of dose.	70% of participants smoked every day. They smoked 1 to 10 joints/day. They used 28 to 56 g/month *[6.5-13 g/week]*.	Not reported
Piper et al. 2017	46% of participants smoked medical cannabis; 23% vaporizing; 14% edibles; 12% tincture.	Vaping: medical cannabis administered with joints was significantly more expensive than via vaporizer.	Smoking: • not always convenient • gross • bad taste. Vaporizing: • cumbersome • too expensive. Edibles: • Lack of availability. Tincture: • takes too long • complex dosing.	Not reported	Decrease in prescription medications.
Reiman 2009	Not reported	Not reported	Not reported	Not reported	66% of participants reported having used cannabis as a substitute for prescription drugs.
Reiman et al. 2017	50% smoking; 30% vaporizing; 10% edibles.	Not reported	Not reported	Not reported	97% of participants decreased the amount of opioids they consume when they also use cannabis. 96% do not need to take as much of their nonopioid-based pain medication when they use cannabis.
Reinarman et al. 2011	86% smoking; 24% orally; 22% vaporizing.	Not reported	Not reported	67% daily use; 53% use 1–2 times per day. ≤3 grams (40%) to ≥7 grams (23%) per week.	50.9% of participants reported use of medical cannabis to substitute prescription medication.
Sagy et al. 2019	Smoking, oil	Not reported	Not reported	From 670 to 1000 mg/day	After 6 months of medical cannabis therapy, a substantial proportion of participants stopped or decreased the dosage of other medical therapies.
Schnelle et al. 1999	49% inhalation; 14% eating, drinking; 36% used both the oral and inhalation routes 4% used dronabinol.	Not reported	Not reported	Not reported	Not reported
Sexton et al. 2016	84% inhalation: 32% pipes, 19% bongs, 16.5% joints/blunts, 16% vaporizer; 8% oral (edibles, tinctures, capsules); 6% concentrates (oil, keif, hash); 0.6% topical; 0.5% fresh juice.	Not reported	Not reported	61% reported using 1–5 hits per smoking session, 21.3% reported 6–10 hits, 18% reported >10 hits/session. 25% reported using less than 1 once/day; 1–4 times/day (47.6%); 5–10 times/day (14.9%), and 12.2% reported using all day, every day. Reported use (g/week): < 1 (12.3%); 1–2 (20.3%); 3–5 (31.8%); 7 (26.1%); 28 (6%), > 28 (3.4%).	60% reported substitute medical cannabis for prescription drugs, 25% for pain medications, including opiates.
Shah et al. 2017	54% smoking; 29% tablets; 8% edibles.	Not reported	Not reported	62.5% of medical cannabis users endorsed daily use, 21% weekly use.	Cannabis use was not associated with a significantly lower morphine equivalence level for participants using prescription opioids.
Shiplo et al. 2016	53% of participants preferred vapourizing; 47% smoking a joint. Among those reporting multiple modes: 25% eating in food, 23% drinking.	• Time to onset of effect. • Ability to find correct dose. • Smoking lower cost and more accessible. • Eating/drinking had more durable effect.	• Harm from smoking. Eating in foods: • producing the worst high • most stigma • hardest to find a correct dose.	Almost every day: 77%, > once a day: 82%. 1.8 ± 1.6 g/day *[12.6 g/week]*.	Not reported
Ste-Marie et al. 2012	Out of the 59 medical cannabis users: 80% smoked herbal cannabis; 24% used prescription cannabinoids; 3% used both.	Not reported	Not reported	72% used < 1 g/day *[< 7 g/week]*.	Not reported
Ste-Marie et al. 2016	86% smoking; 21% vaporizing; 18% ingestion; 4% topical.	Not reported	Not reported	For the 22 patients who recorded amounts used, most reported ≤ 1.5 g/day *[≤ 10.5g/week]*.	Not reported
Swift et al. 2005	91% smoked. 74% considered smoking the most helpful route.	Smoking: • Instant effect. • Ease of titration. • Cost-effectiveness. Edibles: • Healthier • Tasty when cooked in a recipe • Less obvious • Slow onset and long-lasting effects.	Smoking: • Detrimental to respiratory function (and health) Edibles: • Availability of recipes • Difficulties with titration • expensive and ineffective for rapid relief.	75% used at least weekly, 59% used almost daily, 22% used “as required.”	62% of participants claimed they decreased or discontinued their use of other medicines with medical cannabis use.
Troutt & DiDonato 2015	67% inhalation: ~ 42% smoking, ~ 25% vaporizing; ~ 27% edibles; ~ 4% tincture; ~ 2% oils.	Not reported	Not reported	84% used several times per week or more, 61% used daily. 78% used < 14 g/month *[3.2 g/week]*.	90% of chronic pain, 81% of arthritis, 94% of fibromyalgia, and 84% of osteoarthritis patients report less frequent use of other medications.
Walsh et al. 2013	57% smoking; 43% vaporizing; 28% orally.	Not reported	Not reported	53% used 2–3 times/day, 42 used ≥ 4 times/day. 45% used >14 g/week.	
Ware et al. 2003	Among users for pain: 81% joint, 47% joint with tobacco, 34% pipe, 16% water pipe; 9% orally.	Not reported	Not reported	53% used ≤ 4 puffs/dosing interval, 25% smoked a whole cannabis cigarette, 12% smoked ≥ 1 joint. 22% of these participants used cannabis > 1 time/day, 16% used daily, 25% used weekly and 28% rarely used medical cannabis.	Not reported
Webb & Webb 2014	Not reported	Not reported	Not reported	Not reported	6% wrote brief notes relating how cannabis helped them to decrease or to discontinue other medications.
Zaller et al. 2015	74% smoking; 16.5% vaporizing; 7% orally.	Not reported	Not reported	60.5% used ≥ 3 times/day. 48.5% used 3–8 g/day, 34.5% used > 8 g/day *[21–56 g/week]*.	55% indicated they had used cannabis as a substitute for prescription medications.

*1[calculated amount of cannabis use in grams per week]*

*THC*: delta-9-tetrahydrocannabinol

**Table 3:** Perceived positive and negative effects

	Perceived positive effect	Perceived negative effect
Smoking medical cannabis	Enjoyable Easy to titrate Immediate pain relief Less expensive compared to edible or vaping cannabis	Respiratory side effect Bad smell Bad taste
Edible medical cannabis	Healthier Tasty when cooked in a recipe Long-lasting pain relief	
No specific medical cannabis use	Alleviation of pain, headache, anxiety Positive effect on mood Improvement of their general quality of life Longer effect and less adverse effect compare to opioids and other prescription medication	Increase of appetite Drowsiness Cognitive effects Respiratory effects (for cannabis smoking)

### Medical cannabis used as a substitute for prescription medications

Of the 20 studies that examined the impact of MC use on the utilization of other prescribed medications (Boehnke et al., 2016; Swift et al., 2005; Bruce et al., 2018; Haroutounian et al., 2016; Lucas & Walsh, 2017; Lynch et al., 2006; Nunberg et al., 2011; Piper et al., 2017; Reinarman et al., 2011; Sexton et al., 2016; Troutt & DiDonato, 2015; Corroon Jr. et al., 2017; Crowell, 2017; Grella et al., 2014; Reiman, 2009; Reiman et al., 2017; Shah et al., 2017; Webb & Webb, 2014; Zaller et al., 2015; Lavie-Ajayi & Shvartzman, 2018; Sagy et al., 2019), 19 reported that MC consumption was accompanied by a decrease in the number and amount of prescribed drugs used, including opioids, antidepressants, anxiolytics and benzodiazepines, and non-opioid-based pain medication (Boehnke et al., 2016; Swift et al., 2005; Bruce et al., 2018; Haroutounian et al., 2016; Lucas & Walsh, 2017; Lynch et al., 2006; Nunberg et al., 2011; Piper et al., 2017; Reinarman et al., 2011; Sexton et al., 2016; Troutt & DiDonato, 2015; Corroon Jr. et al., 2017; Crowell, 2017; Grella et al., 2014; Reiman, 2009; Reiman et al., 2017; Webb & Webb, 2014; Zaller et al., 2015; Lavie-Ajayi & Shvartzman, 2018; Sagy et al., 2019) (Table 2). In twelve studies, it had been observed that participants discontinued their use of opioids or other prescription drugs following the start of MC consumption (Swift et al., 2005; Bruce et al., 2018; Haroutounian et al., 2016; Lucas & Walsh, 2017; Nunberg et al., 2011; Reinarman et al., 2011; Sexton et al., 2016; Corroon Jr. et al., 2017; Reiman, 2009; Webb & Webb, 2014; Zaller et al., 2015; Lavie-Ajayi & Shvartzman, 2018; Sagy et al., 2019), in a proportion varying from 6% (Webb & Webb, 2014) to 63% of participants (Lucas & Walsh, 2017). Participants also reported preferring the use of MC to prescription medication (Grella et al., 2014), mainly because of the adverse effects of their prescription drugs (Lynch et al., 2006).

### Past and current use of cannabis and other licit and illicit substances

In 18 studies, 20 (Ste-Marie et al., 2016) to 90% (Harris et al., 2000) of participants reported that they had previously consumed cannabis recreationally or that they consumed it simultaneously to their therapeutic cannabis use (Swift et al., 2005; Hazekamp et al., 2013; Ste-Marie et al., 2016; Belle-Isle et al., 2014; Degenhardt et al., 2015; Erkens et al., 2005; Harris et al., 2000; Hoffman et al., 2017; Lucas & Walsh, 2017; Nunberg et al., 2011; Ogborne et al., 2000; Reinarman et al., 2011; Schnelle et al., 1999; Walsh et al., 2013; Ware et al., 2003; Grella et al., 2014; Grotenhermen & Schnelle, 2003; Shah et al., 2017; Lintzeris et al., 2018; Sagy et al., 2019) (Supplemental Table S1). One study reported that 29% of participants discovered the therapeutic effects of cannabis while using it recreationally (Swift et al., 2005). Six studies suggested that there might be a link between current MC use and past consumption of licit and illicit substances, as a proportion of MC users (3 to 89%) reported a past history of substance abuse, including alcohol, cocaine, amphetamines, hallucinogens, or other prescription drugs (Perron et al., 2015; Ilgen et al., 2013; Harris et al., 2000; Bonn-Miller et al., 2014; Grella et al., 2014; Zaller et al., 2015). Moreover, some MC users considered cannabis a substitute for alcohol (up to 26% of participants) (Lucas & Walsh, 2017) or illicit drugs (up to 16% of participants) (Zaller et al., 2015).

### Reported barriers to the medical use of cannabis

Obstacles to the medical use of cannabis have been reported at several levels (Supplemental Table S2), including stigmatization from others (Ogborne et al., 2000; Piper et al., 2017), fear of discrimination (Belle-Isle et al., 2014), and physicians’ unwillingness to recommend, certify, or authorize MC (Aggarwal et al., 2009; Belle-Isle et al., 2014; Lucas & Walsh, 2017; Pedersen & Sandberg, 2013). Some MC users expressed health concerns such as pulmonary health or fear of addiction (Swift et al., 2005; Hoffman et al., 2017; Piper et al., 2017; Grella et al., 2014), but no study explicitly investigated perceived addiction to cannabis as a treatment consequence. Difficulties in finding a consistent and affordable MC supply and fear of legal problems associated with MC consumption, e.g., driving after consumption, represent further obstacles to MC utilization (Swift et al., 2005; Aggarwal et al., 2009; Aggarwal et al., 2013a; Aggarwal et al., 2013b; Belle-Isle et al., 2014; Coomber et al., 2003; Hoffman et al., 2017; Lucas & Walsh, 2017; Ogborne et al., 2000; Piper et al., 2017; Alexandre, 2011; Grotenhermen & Schnelle, 2003; Lintzeris et al., 2018).

## Discussion

### Main findings

In the included studies, a majority of participants who used cannabis for therapeutic purposes to relieve pain were aged 28.4 to 62.8 years in average with a proportion of men varying between 18 and 88% (Table 4). The most frequent mode of cannabis administration was smoking. The majority of MC users consumed cannabis daily, in a quantity ranging between 0.05 and 1 gram/day. MC users from reviewed studies reported positive effects on symptoms alleviation in addition to “secondary outcomes” such as psychological well-being. Reported adverse effects associated with MC utilization were few and of minor intensity and were mainly associated with cannabis smoking, such as negative impacts on pulmonary health. MC users also reported a reduction in the use of prescription drugs for the management of chronic pain (Boehnke et al., 2016; Swift et al., 2005; Bruce et al., 2018; Haroutounian et al., 2016; Lucas & Walsh, 2017; Lynch et al., 2006; Nunberg et al., 2011; Piper et al., 2017; Reinarman et al., 2011; Sexton et al., 2016; Troutt & DiDonato, 2015; Corroon Jr. et al., 2017; Crowell, 2017; Grella et al., 2014; Reiman, 2009; Reiman et al., 2017; Webb & Webb, 2014; Zaller et al., 2015; Lavie-Ajayi & Shvartzman, 2018; Sagy et al., 2019).

**Table 4:**
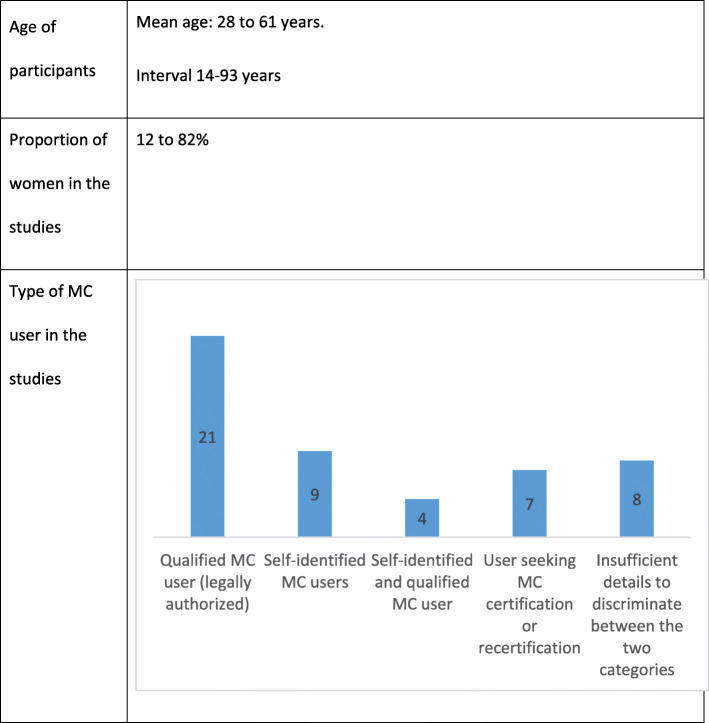
Patients’ characteristics

### Strengths and limitations of the review

To the best of our knowledge, this is the first comprehensive literature review on the perceptions of persons suffering from CMP or other chronic non-cancer pain, who used cannabis for therapeutic reasons. The information gathered in this review represents an opportunity to better understand the perspective of different types of MC users on the multiple dimensions of its consumption, in particular barriers, advantages, and drawbacks.

However, this review has several limitations, related principally to methodological weaknesses in an important proportion of the included studies.

### Selection and recruitment of participants

For 41% of participants, they have been recruited at MC dispensaries, MC associations, or MC advocacy groups, including four studies performed in countries without a legal framework for access to MC (Swift et al., 2005; Coomber et al., 2003; Lintzeris et al., 2018; Pedersen et al., 2016). This might have introduced selection and information biases, as it has been reported that people who are already familiar with cannabis through recreational use, may use cannabis for medical reasons (Bigand et al., 2019; Lum et al., 2019). Indeed, among the about 30% of studies reporting on prior cannabis use, many MC users reported recreational cannabis use prior or simultaneously to MC use. Some MC users reported that it was during the recreational use of cannabis that they discovered its therapeutic effects. Moreover, people who are attending these centers may not use cannabis exclusively for medical reasons. In addition, MC users who had stopped MC consumption participated only marginally in these studies. Prevalence of adverse effects might therefore be underestimated. Furthermore, a subgroup of those studies, for which the source of funding was reported, was financially supported by cannabis interest or patient groups. This may have introduced a positive bias toward the use of cannabis against chronic pain. Therefore, we can argue that study participants were likely not representative of the general population with CMP or other chronic non-cancer pain, since a relevant subgroup of persons suffering from CMP or chronic non-cancer pain, but not considering MC as a therapeutic option, are not represented in the included studies. For instance, the mean age of MC users in the included studies (28.4-62.8 years) was lower than that of patients suffering from CMP, the incidence of which increases with age (Yelin et al., 2016). In addition, overall, the proportion of men in the included studies was higher than that of women, although CMP affects more often women than men (Yelin et al., 2016), suggesting a possible “gender effect”: with cannabis consumption being more popular among men than women (Carliner et al., 2017) and considering that individuals who already have consumed cannabis seem to be more disposed to use it as a therapeutic agent, it is possible that men are more likely to use cannabis for therapeutic purpose than women (Swift et al., 2005).

### Use of MC with a medical prescription, in a dispensary or in self-medication

In addition, in many studies, it was difficult to distinguish between qualified and self-identified MC users, as it was not specified whether MC use was endorsed by a physician-confirmed diagnosis. It was impossible to estimate the prevalence of each type of user in all selected studies and it was thus not possible to estimate the overall prevalence of self-medication in these studies. Prevalence of self-medication is an important aspect, as it is increasing (Park & Wu, 2017), but self-identified MC users may have different characteristics than qualified MC users. It may become important for physicians to consider the possibility of self-medication with cannabis among their patients with CMP or other chronic pain.

### Other methodological concerns

The included studies also varied greatly in terms of objectives, methodology, and participants’ populations, with 13 studies out of 49 (27%) having less than 100 participants. Data obtained during interviews or from questionnaires were self-reported and may suffer from recall or social desirability bias, while chart reviews may not have allowed to capture patient perceptions. The different legal frameworks regarding MC use across the different countries and periods of time might have influenced the availability and quality of MC, the sample size of the studies, and the availability of information on MC users. The conditions permitting to be registered as a MC user as well as access to MC vary between countries, states, and over time. For example, MC can be obtained from pharmacies in the Netherlands (Erkens et al., 2005; Brunt et al., 2014; Hazekamp & Heerdink, 2013), from special dispensaries in some states of the USA (Aggarwal et al., 2009; Aggarwal et al., 2013a; Piper et al., 2017; Troutt & DiDonato, 2015; Bonn-Miller et al., 2014; Grella et al., 2014; Zaller et al., 2015), and since 2013 from registered producers in Canada (Ste-Marie et al., 2016; Lucas & Walsh, 2017; Shiplo et al., 2016), as reflected in the included studies with participants recruited at dispensaries, registration clinics, or through online advertisement.

### Chronic musculoskeletal pain

Although our scoping review aimed to report on MC users dealing with CMP, we identified only two studies that specifically assessed this type of chronic pain (Ste-Marie et al., 2016). The remaining studies comprised various proportions of participants suffering from CMP or non-specified chronic non-cancer pain. This heterogeneity among MC users may have influenced the reported information on MC consumption and its effects, since no distinction has been made relative to participants’ disease. Considering that the pathophysiology of pain varies depending on the syndrome (McMahon et al., 2013), clinical characteristics of participants should be as homogeneous as possible in order to conclude on the effects of MC on participants’ pain perception. It is thus somewhat reassuring that the two articles reporting specifically on patients suffering from CMP observed similar results as the other studies reporting on more heterogeneous populations. Indeed, among 1000 consecutive rheumatology patients, Ste-Marie et al. observed that 28 patients consumed MC. In agreement with the other studies, the authors observed that MC users were younger than the other patients of this clinic (52.8 vs. 62.8 years) and were more likely to be male (*P* = 0.051). In addition, MC users had previously consumed cannabis recreationally and 39.3% of the MC users reported to consume cannabis recreationally, in addition to MC (Ste-Marie et al., 2016).

## Gaps in the literature

We identified some gaps in the literature that need to be addressed to better understand patients’ utilization of MC against MCP and unspecified chronic non-cancer pain. First, future studies should include participants who have stopped MC consumption or do not want to consider it, in order to understand the reasons that lead to discontinuation or rejection of MC, such as stigmatization of cannabis users or onset of adverse effects associated with MC use. As an example, Zolotov et al. reported that among participants who consumed cannabis for medical reasons, including chronic non-cancer pain (47.5%), those who abandoned MC (20%) experienced more frequent adverse effects (dizziness, dehydrated mouth, fatigue, mild anxiety, and feeling “weird”) than those who continued MC use (*P* < 0.05) (Zolotov et al., 2016).

Supported by a recent literature review, it would be interesting to better understand the point of view of physicians to identify the major factors which impact the decision of prescribing or not medical cannabis for patients who suffered from chronic pain (Gardiner et al., 2019). This would bring new knowledge on whether prescribers need support during the informed decision-making regarding the use of MC to treat CMP. The debate among physicians whether or not to prescribe MC is ongoing and has recently been presented in the literature (Caulley et al., 2018). Moreover, a changing legal framework for recreational cannabis may influence the perception of physicians regarding treatment with MC.

The use of MC as a substitute for other drugs, including opioids and other prescription medications, will need to be investigated for improved decision-making regarding MC prescribing, since opioids present serious, well-documented adverse effects. Currently, clinical guidelines in some countries, e.g., Canada, only support the use of MC for specific medical conditions, including neuropathic pain, palliative cancer pain, chemotherapy-induced nausea and vomiting, and spasticity related to multiple sclerosis or spinal cord injury, especially for those patients who do not respond to conventional therapies (Allan et al., 2018). Further randomized clinical trials that evaluate the efficacy and safety of MC in the management of CMP, other chronic pain or as substitute for opioids are urgently needed, but methodological challenges remain, including difficulties in participants’ recruitment and follow-up, and the surveillance of adverse effects.

## Conclusion

Although the included studies are frequently exploratory and might be biased by several factors, they describe the perspective of MC users and allow a better understanding of their attitudes and experiences regarding MC use against CMP and other chronic non-cancer pain. These users perceive MC to have more benefits than drawbacks regarding quality of life and adverse effects, and several report on the possibility that MC might decrease the use of some prescription drugs, particularly opioids. However, these user reported experiences must be examined by well-designed and methodologically sound clinical or observational studies before any conclusions can be drawn.

## Supplementary Information

**Additional file 1.**

## Data Availability

Not applicable.

## Abbreviations

CMP: Chronic musculoskeletal pain
MC: Medical cannabis
THC: Delta-9-tetrahydrocannabinol
CBD: cannabidiol
NNT: Number needed to treat
HIV/AIDS: Human immunodeficiency virus and acquired immune deficiency syndrome
