# Supplementary material for: Cannabis against chronic musculoskeletal pain: a scoping review on users and their perceptions
Source: J Cannabis Res. 2021 Sep 4;3:41. doi: 10.1186/s42238-021-00096-8 (PMC8418709; doi:10.1186/s42238-021-00096-8)
Supplement: Supplementary file 1 — Additional file 1. [file 42238_2021_96_MOESM1_ESM.docx]

**Supplemental table S1: Past and current use of cannabis and other licit and illicit substances**

| **Article** |  | **Source of data** |  | **Recreational use of cannabis** |  | **Use of alcohol or other drugs** |
| --- | --- | --- | --- | --- | --- | --- |
| Bonn-Miller et al. 2014[[16](#_ENREF_16); [23](#_ENREF_23)] |  | 217 patients recruited from a dispensary in California (US). |  | In the overall sample, 21.7% of participants met criteria for cannabis abuse and 16.1% were cannabis dependent.  Rates of cannabis abuse ranged from 15% (among those using for nausea) to 29% (headaches).  Rates of dependence were greatest among those using for muscle spasms and nausea (26 and 25.5%, respectively) and lowest among those using for chronic pain (15%). |  | In the previous 3 months, 14% used hallucinogens; 12% used cocaine; 6% used inhalants; 6% used stimulants; 4% used sedatives and 3% used opiates. |
| Degenhardt et al. 2015[[39](#_ENREF_39)] |  | 1514 Australians who had been prescribed opioids for chronic non-cancer pain. |  | 43% had recreationally used cannabis at some time.  12% had met criteria of cannabis use disorder in their lifetime. |  | Not reported |
| Erkens et al. 2005[[42](#_ENREF_42)] |  | 200 Dutch patients who filled a prescription for medical cannabis. |  | 60% of respondents already used cannabis before the legalization.  80% still obtained medical cannabis from the illegal circuit. |  | Not reported |
| Grella et al. 2014[[50](#_ENREF_50)] |  | 182 medical cannabis dispensary users in California (US). |  | Nearly all participants had a long history of cannabis use prior to receiving their medical cannabis recommendation, usually initiating use in adolescence. |  | In the past 30 days:  46% met the criteria for risky alcohol use;  21% reported use of any illicit drugs or misuse of prescription medications. |
| Grotenhermen & Schnelle, 2003[[51](#_ENREF_51)] |  | 165 members of the Association for Cannabis as Medicine or persons interested in participating (Europe). |  | 48% of the medical cannabis users had experienced cannabis before the onset of their disease. |  | Not reported |
| Harris et al. 2000[[53](#_ENREF_53)] |  | 100 medical cannabis Club members in California (US). |  | 10% had not used cannabis before the onset of their disease. |  | 87% met criteria for lifetime abuse of or dependence on drugs.  17% met criteria for current abuse or dependence. |
| Hazekamp et al. 2013[[55](#_ENREF_55)] |  | 953 participants from 31 countries. |  | 76.5% of participants indicated having used cannabis products prior to the onset of their medical condition. |  | Not reported |
| Hoffman et al. 2017[[57](#_ENREF_57)] |  | 22 cannabis-using patients in Oregon (US). |  | Initiation usually began high school.  5% use cannabis only recreationally, 32% both recreationally and medicinal use. |  | Not reported |
| Ilgen et al. 2013[[59](#_ENREF_59)] |  | 348 patients seeking medical cannabis certification in Michigan (US). |  | Not reported |  | 89% reported alcohol use within their lifetime, 40% reported nonmedical use of opioids in their lifetime.  29% recent abuse of prescription medications; 15% illicit drugs. |
| Lintzeris et al., 2018 |  | 1748 medical cannabis users that participated to an online survey in Australia. |  | 40.8% of participants met the criteria for cannabis use disorder.  55.3% of respondents reported having used cannabis recreationally at the time they commenced medical cannabis use. |  | Not applicable |
| Lucas & Walsh 2017[[64](#_ENREF_64)] |  | 271 patients registered to purchase medical cannabis from a federally authorized licenced producer (Canada). |  | The mean age of initiation was 18.5 ± 7.4 for recreational use and 34.1 ± 13.7 for medical use. |  | 25% reported substitution for alcohol, 3% for illicit substances. |
| Nunberg et al. 2011 and Reinarman et al. 2011[[74](#_ENREF_74); [84](#_ENREF_84)] |  | 1746 MC applicants in California (US). |  | The great majority had used cannabis recreationally, in response to a separate question > 41% reported that they had *not* been using it recreationally prior to trying it for medicinal purposes. |  | 13.2% reported using cannabis as a substitute for alcohol.  Current nonmedical: 57.5% alcohol, 0.4% methamphetamine, 0.3% cocaine, 0.1% heroin, 1.2% other opiates. |
| Ogborne et al. 2000[[75](#_ENREF_75)] |  | 50 self-identified medical cannabis users in Toronto (ON, Canada). |  | 84% had at least some experience with cannabis as a recreational drug, and 60% described a long history of recreational use. medical cannabis use typically started when recreational use was found to be helpful for medical or psychological problems. |  | Not reported |
| Perron et al. 2015[[77](#_ENREF_77)] |  | 273 subjects who reported past-month cannabis use for pain-related purposes. |  | Not reported |  | 14% of the overall sample reached or exceeded the alcohol misuse threshold.  37% reported lifetime use of cocaine, 27% of amphetamines. Past-3-month use: 2.6% cocaine, 3.8% amphetamines. |
| Sagy et al, 2019 |  | 367 fibromyalgia patients that used medical cannabis during a period of 6 months, Israel. |  | 45.2% of participants reported previous use of recreational cannabis in the past. |  | Not applicable |
| Schnelle et al. 1999[[87](#_ENREF_87)] |  | Survey of 128 participants. |  | 52.4% of respondents had experience with cannabis before the disease. |  | Not reported |
| Shah et al. 2017[[89](#_ENREF_89)] |  | 48 medical cannabis patients in a pain rehabilitation program (US). |  | 63% of participants with a positive screen for THC had prior history of recreational use of THC. |  | Participants with a positive screen for THC were more likely to report a past history of illicit substance use and alcohol abuse (29%). |
| Ste-Marie et al. 2016[[93](#_ENREF_93)] |  | 1000 patients with a rheumatologist-confirmed diagnosis. |  | 20% reported past or current recreational use, 2% current recreational use. |  | Not reported |
| Swift et al. 2005[[95](#_ENREF_95)] |  | 128 Australians who had used medical cannabis. |  | 29% had discovered cannabis therapeutic benefits as a spin-off from recreational use.  In addition to medical use, 80% had used cannabis recreationally.  For 46%, use in the past year had been solely medicinal. |  | Not reported |
| Walsh et al. 2013 and Belle-Isle et al. 2014[[19](#_ENREF_19); [101](#_ENREF_101)] |  | 443 participants from medical cannabis dispensaries and from organizations that assist medical cannabis users. |  | 82% reported a history of non-therapeutic prior to therapeutic cannabis use. |  | Not reported |
| Ware et al. 2003[[103](#_ENREF_103)] |  | 209 chronic non-cancer pain patients (Canada). |  | 35% had ever used cannabis. |  | Not reported |
| Zaller et al. 2015[[107](#_ENREF_107)] |  | 200 self-selected patients at 2 Rhode Island (US) compassion centers. |  | Not reported |  | 7.5% ever received treatment for alcohol dependence, 26% ever used cannabis as a substitute for alcohol.  13.5% ever received treatment for illicit drugs, 16% ever used cannabis as a substitute for illicit drugs. |

Abbreviations: THC: delta-9-tetrahydrocannabinol.

**Supplemental Table S2: Reported barriers to the medical use of cannabis**

| **Study** |  | **Stigma** |  | **Experiences with physicians** |  | **Health concerns** |  | **Legality concerns** |  | **Source of cannabis** |  | **Cost of cannabis** |
| --- | --- | --- | --- | --- | --- | --- | --- | --- | --- | --- | --- | --- |
| Aggarwal et al. 2009[[4](#_ENREF_4)]  MC illegal but dispensaries tolerated. |  | Not reported |  | Prior physicians unwilling to authorize. |  | Not reported |  | Legal problems related to medical cannabis use: charges of possession, cultivation, or use of cannabis. |  | Difficulties in finding a consistent supply. |  | Difficulties in finding an affordable supply. |
| Aggarwal et al. 2013a[[3](#_ENREF_3)]  MC illegal but dispensaries tolerated. |  | Not reported |  | Not reported |  | Not reported |  | About 75% from a little bit to moderately stressed regarding federal illegality. |  | Not reported |  | Not reported |
| Alexandre 2011[[7](#_ENREF_7)]  Legal MC use. |  | Not reported |  | 87% reported successful physician encounters; in one case, the primary care physician refused to certify the patient, but the patient was subsequently certified when referred to a specialist for additional treatment. |  | Not reported |  | Feelings of vulnerability, paranoia, and of participating in criminal behavior prior to enrollment.  No indication that patients had been arrested or prosecuted for participating in the program. |  | The initial iteration provided patients no legal access to marijuana. Registered patients were expected to grow their own or appoint someone to assist them. In response to patient concerns, the act was revised the act in 2009. |  | Concerns with respect to cost, growing, quality, availability of specific strains, statutory limits on the quantity. |
| Belle-Isle et al. 2014[[19](#_ENREF_19)]  Legal MC. |  | Reported reasons for respondents avoiding medical cannabis in the past included: “I could be discriminated against” (60%). |  | 48% reported they had wanted to discuss medical cannabis with a physician but had not done so. 38% had not discussed medical cannabis with any physician: “don’t feel comfortable” (62%).  32% sought another physician in relation to use of medical cannabis, 57% of whom changed physicians more than once.  29% reported that physician recommended medical cannabis but refused to endorse application for authorized access. |  | NR |  | 16% of self-producers reported arrests, 12% reported break ins.  32% of medical cannabis users who do not self-produce had legal concerns.  Illegality was among the reasons for not discussing medical cannabis with a physician (46%). |  | 31% authorized source,  76% of them also accessed medical cannabis from unauthorized sources.  31% reported self-producing for personal use, 39% of them did so for quality, 24% for a specific strain. 34% found difficult to learn to cultivate cannabis. |  | Among reasons for not discussing medical cannabis with a physician: “can’t afford cannabis” (9%).  40% of applicants were charged by physicians for having their application completed ($10 to $800).  36% of self-producers do so to reduce expenses. 37% of medical cannabis users who do not self-produce found the set up too expensive.  54% of respondents reported they were sometimes or never able to afford to buy sufficient quantity to relieve their symptoms. |
| Bottorff et al. 2011[[24](#_ENREF_24)]  Legal medical cannabis. |  | Not reported |  | Not reported |  | In general, participants were not overtly concerned with health or dependence risks. |  | Not reported |  | Health risks posed by unregulated sources: unknown strain and quality. |  | Not reported |
| Coomber et al. 2003[[32](#_ENREF_32)]  No legal access to medical cannabis then. |  | Cannabis use was not perceived as causing problems between the interviewees and their family members. |  | 33% told their health care management their medical cannabis use.  Reactions reported as being either favorable (51%), having turned a “deaf ear” (6%), or as mixed. |  | Not reported |  | Illegality of cannabis had no impact on their decision to use it (45%) or was appealing (21%). |  | 55% either bought the drug themselves, grew it, or both. |  | 42% deemed the expense problematic. |
| Grella et al. 2014[[50](#_ENREF_50)]  Legal medical cannabis. |  | Not reported |  | Not reported |  | Two participants reflect on whether their use of medical cannabis was an “addiction.” |  | Not reported |  | Participants were unanimous in their preference for obtaining marijuana from legal dispensaries as compared to buying it “from a dealer on the streets.” |  | Not reported |
| Grotenhermen & Schnelle 2003[[51](#_ENREF_51)]  Canabis illegal. |  | Not reported |  | 86% had asked their doctor to prescribe THC. In 54.8% the doctor was willing to do so, but in 55% of the cases, the health insurance companies refused to pay for the treatment. |  | 22 non users because the unavailability of studies on the safety of use. |  | 41% of non-users feared criminal prosecution.  22% of medical cannabis users feared criminal involvement. |  | Non users:  Fear of meeting the wrong people when buying cannabis.  No serious supply with constant quality. |  | Non users: cost |
| Hoffman et al. 2017[[57](#_ENREF_57)]  Legal cannabis. |  | Not reported |  | Not reported |  | No respondent felt having an addiction but 9% feared it.  Most of the smokers expressed concern about pulmonary health. |  | Not reported |  | Not reported |  | 45% had a medical cannabis card. Of those who did not, some expressed that they would like to pursue that option but could not afford to pay for the fees associated with the procurement of the card such as the medical consultation. |
| Lintzeris et al., 2018  medical cannabis illegal. |  | 47.4% of respondents were concerned about employment security because of medical cannabis use. |  | Not reported |  | Not reported |  | 84.9% of participants reported being worried about being arrested or other legal problems. |  | Concerns about unconstant Not reportedquality (52%) |  | Concerns with respect to cost (56.2%). |
| Lucas & Walsh 2017[[64](#_ENREF_64)]  Legal medical cannabis. |  | Not reported |  | Finding a supportive physician was a reported challenge with 31% having changed doctors in relation to medical cannabis use, and 55% reporting feeling discriminated against by their doctor because of medical cannabis use. |  | Not reported |  | Not reported |  | 21% also reported purchasing from another Licensed Producer, 25% from dispensaries, 18% from a friend, and 8% buy from an illicit dealer. In total, 42% reported accessing from ≥1 unregulated sources. |  | Capacity to “often” or “always” afford to buy enough cannabis to relieve symptoms was reported by 40%, leaving 60% who report “sometimes” or “never” affording sufficient cannabis.  Paying a physician or clinic for recommendations to use medical cannabis reported by 55%. |
| Ogborne et al. 2000[[75](#_ENREF_75)]  No legal access to medical cannabis then. |  | Discomfort with the smell.  Rejection by family members. |  | 76% told their doctors about their medical cannabis use.  Most found doctors noncommittal or supportive but, for 8%, doctors discouraged them from using cannabis. |  | None of the respondents expressed significant concerns regarding cannabis dependence. |  | That cannabis is an illegal drug weighed on the minds of most respondents.  ~ 30% of those interviewed reported being stopped and searched for cannabis or other drugs and drug paraphernalia at some time. |  | Almost all respondents obtained cannabis from regular dealers or friends. |  | Having to buy on the street resulted in a financial burden for most interviewees. |
| Pedersen & Sandberg 2013[[76](#_ENREF_76)]  No legal access to medical cannabis then. |  | Not reported |  | Many medical cannabis users described spending years in negotiation with their general practitioners. Most were sceptical and even those supportive did no more than refer the patients to another medical professional. |  | Not reported |  | Not reported |  | Not reported |  | Not reported |
| Piper et al. 2017[[79](#_ENREF_79)]  Legal medical cannabis. |  | 11.4% view of others |  | Not reported |  | Perceived limited addictive potential of cannabis.  Effects on lungs, appetite, cognition (non-alert feeling, hangover) and occasional paranoia/ anxiety. |  | 6.2%: differences between federal and state laws (crossing boarders).  Inconvenience of dosing in secret. |  | 8% access: dispensary location and characteristics (hours, strain availability). |  | 28.4% economic considerations. |
| Swift et al. 2005[[95](#_ENREF_95)]  No legal access to medical cannabis then. |  | Family and friends were largely considered supportive of the participant's use (71%). |  | 90% had informed a clinician of their therapeutic use, typically reporting a supportive response from general practitioners (75%), specialists (74%) and nurses (81%). |  | 32% concerned over potential health effects.  21% concerned over the risk of dependence. |  | Illegal status 76%, fear of being arrested 60%. |  | Of those who have stopped medical cannabis use, 47% were unable to obtain regular supply.  Of those using intermittently, many reported their use would be more regular if it were more readily availability. |  | 51% concerned about the cost.  37% of those who have stopped did so because of the cost.  Of those using intermittently, many reported their use would be more regular if it were cheaper. |
| Troutt & DiDonato 2015[[99](#_ENREF_99)]  Legal medical cannabis. |  | Not reported |  | Not reported |  | Not reported |  | 89% reported that acquiring medical cannabis was safer after legalization. |  | 85% more confident medical cannabis was safer (less contamination) after legalization. |  | Not reported |
